# The clinicopathological characteristics of POLE-mutated/ultramutated endometrial carcinoma and prognostic value of POLE status: a meta-analysis based on 49 articles incorporating 12,120 patients

**DOI:** 10.1186/s12885-022-10267-2

**Published:** 2022-11-10

**Authors:** Qing Wu, Nianhai Zhang, Xianhe Xie

**Affiliations:** 1 Department of Oncology, Molecular Oncology Research Institute, The First Affiliated Hospital, Fujian Medical University, Chazhong Road No. 20, Fujian 350005 Fuzhou, China; 2 Department of Oncology, National Regional Medical Center, Binhai Campus of the First Affiliated Hospital, Fujian Medical University, Fuzhou, 350212 China; 3 Fujian Key Laboratory of Precision Medicine for Cancer, The First Affiliated Hospital, Fujian Medical University, Fuzhou, 350005 China

**Keywords:** POLE-mutated/ultramutated, Endometrial carcinoma, Overall survival, Progression free survival, Disease specific survival, Relapse free survival

## Abstract

**Objective:**

This study was designed to investigate the frequency and clinicopathological characteristics of POLE-mutated/ultramutated (POLEmut) in endometrial carcinoma (EC) and assess the prognostic values of POLE status.

**Methods:**

Electronic databases were screened to identify relevant studies. Meta-analysis was used to yield the pooled frequency of POLEmut and prognostic parameters by 95% confidence interval (CI), odd ratio (OR), and hazard ratio (HR).

**Results:**

Totally, 12,120 EC patients from 49 studies were included. The pooled frequency of POLEmut was 7.95% (95% CI: 6.52–9.51%) in EC, 7.95% (95% CI: 6.55–9.46%) in endometrioid endometrial carcinoma, and 4.45% (95% CI: 2.63–6.61%) in nonendometrioid endometrial carcinoma. A higher expression occurred in grade 3 (OR = 0.51, 95% CI: 0.36–0.73, *P* = 0.0002), FIGO stage I-II (OR = 1.91, 95% CI: 1.29–2.83, *P* = 0.0013), and myometrial invasion< 50% (OR = 0.66, 95% CI: 0.50–0.86, *P* = 0.0025). Survival analyses revealed favorable OS (HR = 0.68, 95% CI: 0.55–0.85, *P* = 0.0008), PFS (HR = 0.74, 95% CI: 0.59–0.93, *P* = 0.0085), DSS (HR = 0.61, 95% CI: 0.44–0.83, *P* = 0.0016), and RFS (HR = 0.47, 95% CI: 0.35–0.61, *P* <  0.0001) for POLEmut ECs. Additionally, the clinical outcomes of POLEmut group were the best, but those of p53-abnormal/mutated (p53abn) group were the worst, while those of microsatellite-instable (MSI)/hypermutated group and p53-wild-type (p53wt) group were medium.

**Conclusions:**

The POLEmut emergered higher expression in ECs with grade 3, FIGO stage I-II, and myometrial invasion< 50%; it might serve as a highly favorable prognostic marker in EC; the clinical outcomes of POLEmut group were the best one among the four molecular subtypes.

**Supplementary Information:**

The online version contains supplementary material available at 10.1186/s12885-022-10267-2.

## Introduction

Endometrial carcinoma (EC) is one of the most prevalent among gynecological cancer with a steady increase in incidence worldwide [1, 2]. Histotype and other clinicopathological parameters [such as Federation International of Gynecology and Obstetrics (FIGO) stage and tumor grade] are associated with the prognosis of ECs [3, 4]. However, both histotype and grade assignment are relatively poor reproducible [5–7], which leads to inaccurate findings within clinical trials, and over- or undertreatment of ECs.

In order to improve the clinical/pathology-based risk stratification system, the updated classification of EC identifies four subtype [polymerase-ε-mutated/ultramutated (POLEmut), microsatellite-instable (MSI)/hypermutated or mismatch repair-deficient (MMRd), p53-wild-type (p53wt), and p53-abnormal/mutated (p53abn)] according to The Cancer Genome Atlas (TCGA) and Proactive Molecular Risk Classifier for Endometrial Cancer (ProMisE) based on various genetic and molecular features possesses a potential promise, proving to be reproducible, and demonstrating the associations with clinical outcomes [8–11].

POLE is involved in DNA replication and has recently been recognized as hereditary cancer-predisposing genes. The alterations of POLE are associated with occurrence, development and prognosis of tumors, especially in EC [12]. The group of POLEmut, ECs with mutations in DNA POLE that is responsible for DNA replication and leads to exceedingly high somatic mutation frequencies (“ultramutated”: > 100 mutations per megabase) [13, 14], was found to be associated with markedly favorable outcomes, even with poor clinicopathological features [15, 16]. Additionally, they were also candidates for therapy of immune checkpoint inhibitor (ICIs) [17, 18].

However, a consensus has not been reached, with some studies advocating non-superior survival in POLEmut ECs [19, 20]; additionally, the frequency and specific clinicopathological features of POLEmut ECs were various in different studies. Therefore, it remains to be fully illuminated the histopathological features and prognostic of POLEmut ECs. Previous study had preliminarily explored the POLEmut ECs through meta-analysis [21], but it was based on limited histopathological features and prognostic parameters. Consequently, we made a comprehensive survey based on a large scale (49 articles incorporating 12,120 EC patients), multi-level (including eight subgroup analyses), and diverse dimensions (incorporating overall survival (OS), progression free survival (PFS), disease specific survival (DSS), and relapse free survival (RFS)) to summarize the pooled frequency and clinicopathological characteristics of POLEmut ECs and to assess the prognostic value.

## Materials and methods

### Data sources and literature searches

Studies were screened by a systematic electronic literature retrieval for abstracts of relevant studies in the published literature. PubMed, Cochrane Library, and EMBASE were searched and the data were updated as of December 30th, 2021. The basic search terms were used as follows: “endometrial carcinoma”, “endometrial cancer”, “POLE”, “polymerase epsilon”, and “Polymerase ɛ”. Full-text papers were scrutinized if abstracts did not provide substantial information. Moreover, the references of relevant articles were reviewed for additional studies. Data retrieval was completed in English.

### Selection of studies and definition

Initially, two investigators performed a screening of titles and abstracts respectively, then examined the full-text of articles to acquire eligible studies. For the duplicate studies based on the same study patients, only the latest or most comprehensive data were included.

OS was defined as time from surgery until death of any cause; PFS was defined as time from surgery until there is evidence of progressive disease or if they died of the disease prior to the censoring date; DSS was defined as time from surgery until death due to EC; RFS was defined as time from surgery until there is evidence of recurrent disease.

### Inclusion criteria

(1) Prospective or retrospective studies to report the frequency and clinicopathological characteristics of POLEmut in EC; (2) the expression of POLE gene was reported using genetic testing (e.g. sequencing, sanger sequencing, next generation sequencing, and polymerase chain reaction); (3) a full paper had been published.

### Data extraction

Data extraction was implemented conforming to the PRISMA guidance (Table S1). All eligible studies involved information as follows: the publication year and country, first author’s name, study type, and number of both ECs and POLEmut ECs.

### Quality assessment

The quality of included studies was assessed independently by two reviewers using the Newcastle-Ottawa Scale (NOS) for case-control and cohort studies, which encompassed the three dimensions of selection, comparability, and exposure, with a full score of 9 points.

### Statistical methods

The primary endpoint was to report the pooled frequency of POLEmut in ECs. Subgroup analyses were accomplished based on histotype, grade, FIGO stage, lymphovascular space invasion (LVSI), myometrial invasion, lymph node status, clinical risk stratification and adjuvant therapy. The measures to summarize them were odd ratios (ORs) and 95% confidence intervals (CIs). The second endpoint was to evaluate the prognostic value (including OS, PFS, DSS, and RFS) of POLEmut in ECs. The summary measures of survival analysis were hazard ratios (HRs) with corresponding 95% CIs. Funnel plots and Egger’s test were implemented to evaluate publication bias. Statistical analysis was performed through R 4.0 statistical software. Heterogeneity was assessed by I-square tests and chi-square. If *P* <  0.1 or *I*^2^ > 40%, remarkable heterogeneity existed. A random effect model was adopted to analysis the pooled data when heterogeneity existed, otherwise, a fixed effect model was employed.

## Results

### Selection of study

**Fig. 1:**
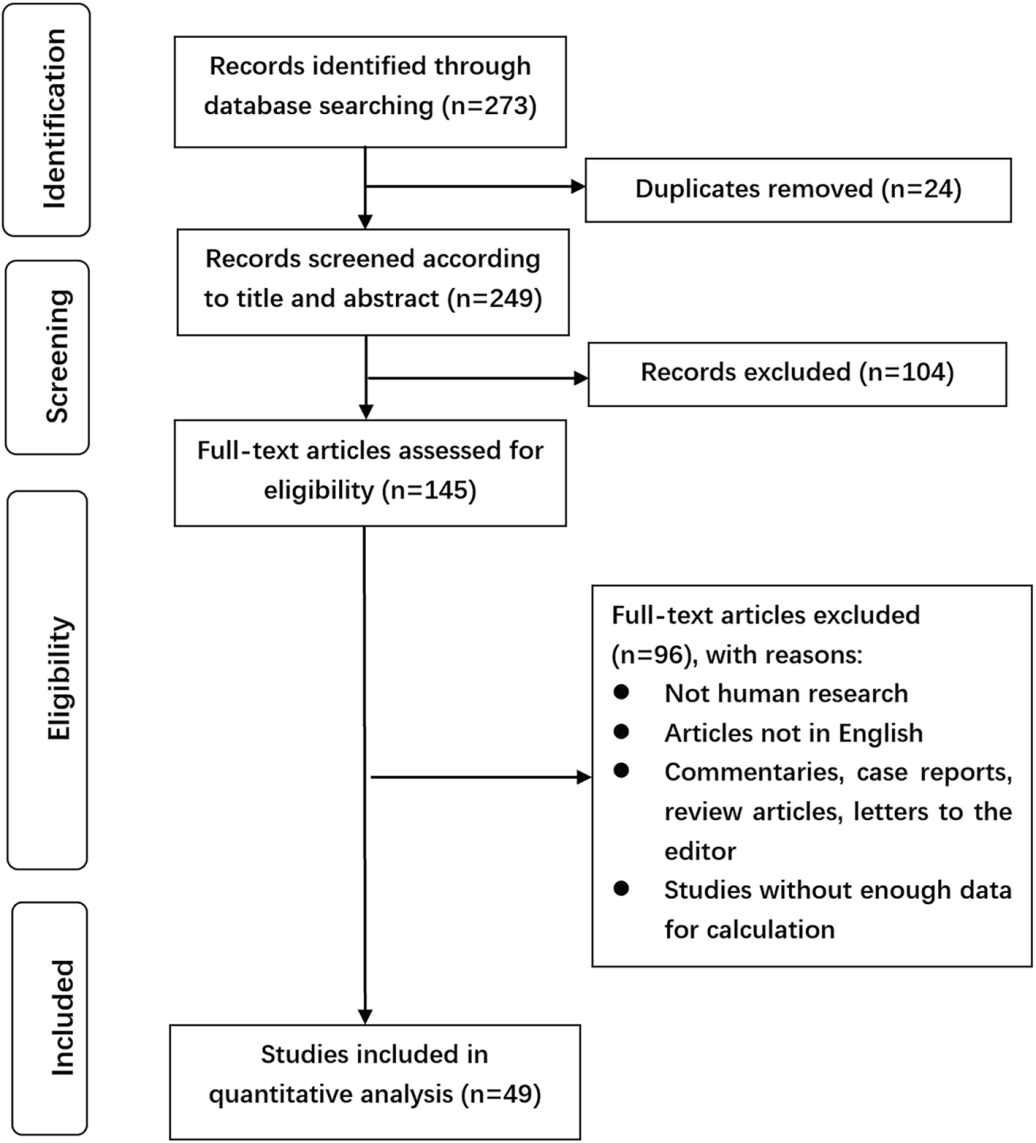
Flowchart on selection including trials in the meta-analysis

Initially, 273 relevant articles were scrutinized intensively. Of them, 24 were filtered for duplication, and 104 were excluded for digression after screening the titles and abstracts. Then the full text of 145 articles was thoroughly reviewed, and 96 were filtered for: they were not human research, and not in English, commentaries, case reports, review articles, letters to the editor, and studies without enough data for calculation. Finally, a total of 49 articles (Table S2) incorporating 12,120 patients were included in this study. The elaborate procedure was displayed in Fig. 1.

### Study traits

Totally, 12,120 individuals in the 49 articles (50 cohorts) published until December 30th, 2021 were included. Studies were published from 2013 to 2021. The sample size ranged from 14 to 982. Of these studies, 8 were prospective, and 41 were retrospective. ORs and 95% CIs were used to report the frequency and clinicopathological characteristics of POLEmut in ECs, and HRs with corresponding 95% CIs were utilized to assess the value of POLEmut in clinical prognosis. Of all the adopted studies, 16 cohorts contained data for OS, 10 for PFS, 8 for DSS, and 8 for RFS. The principal characteristics were listed in Table 1.

**Table 1:** The principal characteristics and further details of eligible articles

Author	Year	Country	Study type	EC size	POLEmut	MSI	p53abn	Sequencing method	Histotype	Location of exonuclease mutations	Outcomes
Abdulfatah et al	2019	USA	retrospective	60	2	20	9	Sanger sequencing	EEC(39); NEEC(21)	Exons 9 and 13	NA
Beinse et al	2020	France	prospective	125	4	35	30	Sequencing	EEC(103); NEEC(22)	NA	NA
Bellone et al	2017	USA and Italy	retrospective	131	11	NA	NA	Sequencing	EEC(96); NEEC(35)	Exons 9–14	OS
Billingsley et al	2015	USA	prospective	544	30	NA	NA	Sanger sequencing	EEC(544); NEEC(0)	Residues 268-471	OS; PFS
Bosquet et al	2021	USA	retrospective	239	28	67	70	Sequencing	EEC(192); NEEC(47)	NA	PFS
Bosse et al	2018	USA, Canada, and Europe	retrospective	376	48	136	79	Sanger or next-generation approaches	EEC(376); NEEC(0)	Exons 9–14	OS; RFS
Church et al	2014	Europe	retrospective	788	48	NA	NA	Sanger sequencing	EEC(770); NEEC(18)	Exons 9 and 13	OS; DSS; RFS
Church et al	2013	Europe	retrospective	173	14	24	NA	Sequencing	EEC(154); NEEC(19)	residues 268–471	NA
Conlon et al	2020	USA	retrospective	37	4	6	NA	Sanger sequencing	EEC(0); NEEC(37)	Exons 9, 13 and 14	NA
Cosgrove et al	2018	USA	prospective	982	39	379	84	Sequencing	EEC(982); NEEC(0)	Exons 9, 13 and 14	OS; PFS; DSS
Crumley et al	2019	USA	retrospective	132	1	NA	NA	Next generation sequencing	EEC(132); NEEC(0)	Exons 9–14	NA
Dai et al	2021	NA	retrospective	473	73	148	170	Sequencing	EEC(363); NEEC(110)	NA	NA
DeLair et al	2017	USA	retrospective	30	2	4	8	Sequencing	EEC(0); NEEC(30)	Exons 9–14	NA
Devereaux et al	2021	USA	prospective	310	15	79	81	SNaPshot technique	EEC(220); NEEC(90)	Exons 9, 11, 13 and 14	NA
Eggink et al	2017	Europe and Australia	retrospective	116	15	19	40	Sanger sequencing	EEC(86); NEEC(30)	Exons 9, 13 and 14	NA
Espinosa et al	2017	Spain	retrospective	21	9	5	NA	Sequencing	NA	Exons 9–14	NA
Espinosa et al	2016	Spain	retrospective	20	1	NA	4	Sequencing	EEC(0); NEEC(20)	Exons 13 and 14	NA
van Esterik M et al	2017	Netherlands	retrospective	49	10	11	10	Sanger sequencing	EEC(42); NEEC(7)	Exons 9 and 13	NA
Falcone et al	2019	Italy	retrospective	15	3	4	1	Sequencing	EEC(15); NEEC(0)	NA	NA
Le Gallo M et al	2017	USA and Europe	retrospective	63	0	7	25	Sanger sequencing	EEC(0); NEEC(63)	NA	NA
Haraldsdottir et al	2014	USA	retrospective	14	3	NA	NA	Next generation sequencing	EEC(11); NEEC(3)	NA	NA
Haruma et al	2018	Japan	retrospective	138	12	40	NA	Sanger sequencing	EEC(123); NEEC(15)	Exons 9 and 13	NA
He et al	2020	China	retrospective	426	38	94	77	Sanger sequencing	EEC(364); NEEC(62)	Exons 9, 13 and 14	OS; PFS
Hoang et al	2015	Canada	retrospective	14	1	NA	4	Sanger sequencing	EEC(0); NEEC(14)	Exons 9–14	NA
Imboden et al	2019	Sweden	retrospective	599	38	NA	NA	Sanger sequencing	EEC(499); NEEC(100)	Exons 9–14	OS; PFS; DSS; RFS
Joehlin-Price et al	2021	USA	retrospective	95	10	35	18	Next generation sequencing	EEC(95); NEEC(0)	Exons 9, 13 and 14	OS; RFS
Jones et al	2020	USA	retrospective	621	28	203	NA	Next generation sequencing	EEC(621); NEEC(0)	NA	NA
Kim et al	2020	Canada	retrospective	52	1	5	18	Sequencing	EEC(0); NEEC(52)	NA	OS; PFS; DSS
Kolehmainen et al	2021	Finland	retrospective	604	30	287	69	Sequencing	EEC(535); NEEC(69)	Exons 9, 13 and 14	NA
León-Castillo et al	2020	UK, Italy, Canada, France, Australia, New Zealand	retrospective	410	51	137	93	Sequencing	EEC(274); NEEC(136)	Exons 9–14	OS; RFS
Li et al	2020	USA	retrospective	529	55	NA	NA	Sanger sequencing	EEC(396); NEEC(133)	Exons 9, 13 and 14	NA
		China	retrospective	467	34	NA	NA	Sanger sequencing	EEC(398); NEEC(69)	Exons 9, 13 and 14	NA
López-Reig et al	2019	Spain	prospective	96	16	NA	32	Next generation sequencing	EEC(83); NEEC(13)	NA	OS; RFS
McConechy et al	2016	Canada	retrospective	406	39	NA	NA	Sequencing	EEC(315); NEEC(91)	Exons 9–14	OS; DSS; PFS
Meng et al	2014	Canada	retrospective	102	16	NA	NA	Sanger sequencing	EEC(102); NEEC(0)	Exons 9–14	OS; PFS; DSS
Monsur et al	2021	Japan	retrospective	127	5	NA	NA	Sequencing	EEC(109); NEEC(18)	NA	NA
Da Cruz Paula A et al	2021	USA	retrospective	175	12	49	39	Sequencing	EEC(116); NEEC(59)	NA	NA
Prendergast et al	2019	USA	retrospective	74	1	13	32	Sequencing	EEC(38); NEEC(36)	NA	NA
Riggs et al	2020	Caucasian, African American, Asian	prospective	65	28	7	NA	Sequencing	EEC(37); NEEC(28)	NA	NA
Rosa-Rosa et al	2016	USA and Europe	retrospective	18	2	8	2	Sanger sequencing	EEC(0); NEEC(18)	Exons 9 and 13	NA
Siraj et al	2019	Riyadh, Saudi Arabia	retrospective	414	2	52	NA	Capture sequencing and Sanger sequencing	EEC(370); NEEC(50)	NA	NA
Stasenko et al	2020	USA	prospective	451	23	NA	NA	Sequencing	EEC(451); NEEC(0)	residues 268–471	NA
Talhouk et al	2015	Canada	retrospective	143	12	41	25	Sanger sequencing	EEC(119); NEEC(24)	Exons 9–14	OS; DSS; RFS
Talhouk et al	2017	Canada	retrospective	319	30	64	86	Sanger sequencing	EEC(215); NEEC(104)	Exons 9–14	OS; PFS; DSS
Tessier-Cloutier et al	2021	Canada, USA, Australia	retrospective	82	6	52	NA	Sequencing	EEC(0); NEEC(82)	Exons 9–14	NA
Cancer Genome Atlas Research Network et al	2013	USA	retrospective	232	17	65	60	Exome sequencing	NA	Pro286Arg and Val411Leu	PFS
Timmerman et al	2020	Belgium	prospective	108	7	33	24	Sanger sequencing	EEC(87); NEEC(21)	Exons 9, 11, 13 and 14	NA
Wong et al	2016	Singapore	retrospective	47	14	20	NA	Next generation sequencing	EEC(47); NEEC(0)	Exons 9–14	OS. RFS
ZHANG et al	2021	China	retrospective	21	3	11	6	Sanger sequencing	NA	Exons 9–14	NA
Zong et al	2021	China	retrospective	587	49	163	130	Sequencing	EEC(594); NEEC(239)	Exons 9–14	NA

Author	FIGO stage				Grade			LVSI		Myometrial invasion		Lymph node status		Clinical risk stratification			Adjuvant therapy	
	I	II	III	IV	G1	G2	G3	present	absent	≥50%	<50%	present	absent	low	intermediate	high	Yes	No
Abdulfatah et al	46	5	9	0	19	22	10	NA	NA	NA	NA	NA	NA	NA	NA	NA	NA	NA
Beinse et al	84	2	26	9	61	29	13	29	87	NA	NA	NA	NA	40	21	40	NA	NA
Bellone et al	62	23	34	12	16	42	73	NA	NA	NA	NA	NA	NA	NA	NA	NA	102	29
Billingsley et al	NA	NA	NA	NA	267	NA	NA	181	343	157	336	NA	NA	NA	NA	NA	370	162
Bosquet et al	NA	NA	NA	NA	72	73	47	NA	NA	NA	NA	NA	NA	NA	NA	NA	NA	NA
Bosse et al	291	NA	NA	NA	0	0	376	NA	NA	NA	NA	NA	NA	NA	NA	NA	NA	NA
Church et al	742	46	0	0	571	108	109	70	718	560	228	NA	NA	NA	NA	NA	576	212
Church et al	114	18	15	8	64	59	45	NA	NA	NA	NA	NA	NA	NA	NA	NA	NA	NA
Conlon et al	19	1	11	6	NA	NA	NA	NA	NA	NA	NA	NA	NA	NA	NA	NA	NA	NA
Cosgrove et al	732	91	141	18	407	423	152	227	737	260	537	NA	NA	NA	NA	NA	NA	NA
Crumley et al	112	5	13	2	NA	NA	NA	30	102	30	102	11	77	NA	NA	NA	NA	NA
Dai et al	NA	NA	NA	NA	NA	NA	NA	NA	NA	NA	NA	NA	NA	NA	NA	NA	NA	NA
DeLair et al	15	0	2	13	NA	NA	NA	NA	NA	NA	NA	NA	NA	NA	NA	NA	NA	NA
Devereaux et al	177	12	66	24	NA	NA	32	99	167	115	104	NA	NA	NA	NA	NA	NA	NA
Eggink et al	42	21	41	11	13	5	98	55	40	87	23	NA	NA	0	0	116	82	10
Espinosa et al	10	1	5	5	NA	NA	NA	NA	NA	NA	NA	NA	NA	NA	NA	NA	12	6
Espinosa et al	11	2	5	2	NA	NA	NA	7	13	NA	NA	NA	NA	NA	NA	NA	16	4
van Esterik M et al	NA	NA	NA	NA	NA	NA	NA	7	42	22	27	NA	NA	17	19	13	NA	NA
Falcone et al	0	0	0	0	14	1	0	NA	NA	NA	NA	NA	NA	NA	NA	NA	NA	NA
Le Gallo M et al	NA	NA	NA	NA	NA	NA	NA	NA	NA	NA	NA	NA	NA	NA	NA	NA	NA	NA
Haraldsdottir et al	12	0	2	0	6	3	5	NA	NA	NA	NA	NA	NA	NA	NA	NA	NA	NA
Haruma et al	93	11	24	10	64	29	45	40	98	49	89	NA	NA	NA	NA	NA	74	64
He et al	NA	NA	NA	NA	NA	NA	108	48	378	117	309	22	345	NA	NA	NA	NA	NA
Hoang et al	6	4	3	1	NA	NA	NA	NA	NA	NA	NA	NA	NA	NA	NA	NA	NA	NA
Imboden et al	447	55	70	27	NA	NA	166	162	437	236	309	63	237	238	70	203	84	258
Joehlin-Price et al	NA	NA	NA	NA	0	0	95	NA	NA	NA	NA	NA	NA	NA	NA	NA	40	55
Jones et al	NA	NA	NA	NA	113	172	156	NA	NA	NA	NA	NA	NA	NA	NA	NA	NA	NA
Kim et al	30	5	14	3	NA	NA	NA	35	16	13	31	9	25	NA	NA	NA	25	20
Kolehmainen et al	440	47	97	20	293	155	87	160	444	249	355	NA	NA	NA	NA	NA	NA	NA
León-Castillo et al	127	105	178	0	NA	NA	113	255	155	NA	NA	223	187	0	0	410	410	0
Li et al	330	51	121	27	96	116	295	NA	NA	NA	NA	NA	NA	NA	NA	NA	NA	NA
	388	37	38	4	321	58	63	NA	NA	NA	NA	NA	NA	NA	NA	NA	NA	NA
López-Reig et al	NA	NA	NA	NA	45	28	23	NA	NA	NA	NA	NA	NA	NA	NA	NA	64	32
McConechy et al	282	28	64	25	125	70	205	NA	NA	NA	NA	NA	NA	NA	NA	NA	180	220
Meng et al	29	6	23	12	0	0	102	25	53	NA	NA	NA	NA	NA	NA	NA	NA	NA
Monsur et al	81	22	21	3	70	23	16	NA	NA	NA	NA	NA	NA	NA	NA	NA	NA	NA
Da Cruz Paula A et al	129	6	30	10	71	35	10	NA	NA	NA	NA	NA	NA	NA	NA	NA	NA	NA
Prendergast et al	12	7	28	24	12	15	44	NA	NA	NA	NA	NA	NA	NA	NA	NA	NA	NA
Riggs et al	31	5	14	12	20	12	33	NA	NA	NA	NA	NA	NA	NA	NA	NA	NA	NA
Rosa-Rosa et al	6	1	4	4	NA	NA	NA	NA	NA	NA	NA	NA	NA	NA	NA	NA	NA	NA
Siraj et al	267	46	66	34	140	138	123	88	233	NA	NA	NA	NA	NA	NA	NA	NA	NA
Stasenko et al	NA	NA	NA	NA	NA	NA	NA	NA	NA	NA	NA	NA	NA	NA	NA	NA	NA	NA
Talhouk et al	102	NA	NA	NA	51	39	53	58	79	NA	NA	19	120	56	23	64	63	79
Talhouk et al	221	NA	NA	NA	NA	NA	196	113	189	118	145	19	150	95	49	173	147	163
Tessier-Cloutier et al	35	5	22	20	NA	NA	NA	NA	NA	NA	NA	NA	NA	NA	NA	NA	NA	NA
Cancer Genome Atlas Research Network et al	NA	NA	NA	NA	NA	NA	NA	NA	NA	NA	NA	NA	NA	NA	NA	NA	NA	NA
Timmerman et al	76	8	18	6	61	17	30	28	79	NA	NA	11	66	44	17	14	37	71
Wong et al	24	6	10	5	0	0	47	29	18	NA	NA	NA	NA	NA	NA	NA	NA	NA
ZHANG et al	13	0	7	0	NA	NA	NA	NA	NA	NA	NA	NA	NA	NA	NA	NA	NA	NA
Zong et al	543	39	173	44	NA	NA	454	231	582	219	585	125	497	NA	NA	NA	NA	NA

Note: The details of included studies can be found in the Table S2.

*Abbreviations*: *EC* endometrial carcinoma, *POLE* Polymerase ɛ, *POLEmut* POLE-mutated/ultramutated, *MSI* microsatellite-instable/hypermutated, *p53abn* p53-abnormal/mutated, *NA* not available, *EEC* endometrioid endometrial carcinoma, *NEEC* nonendometrioid endometrial carcinoma, *OS* overall survival, *PFS* progression free survival, *DSS* disease specific survival, *RFS* relapse free survival, *FIGO* Federation International of Gynecology and Obstetrics, *LVSI* lymphovascular space invasion

### Data analyses

#### The frequency of POLEmut in EC

**Fig. 2:**
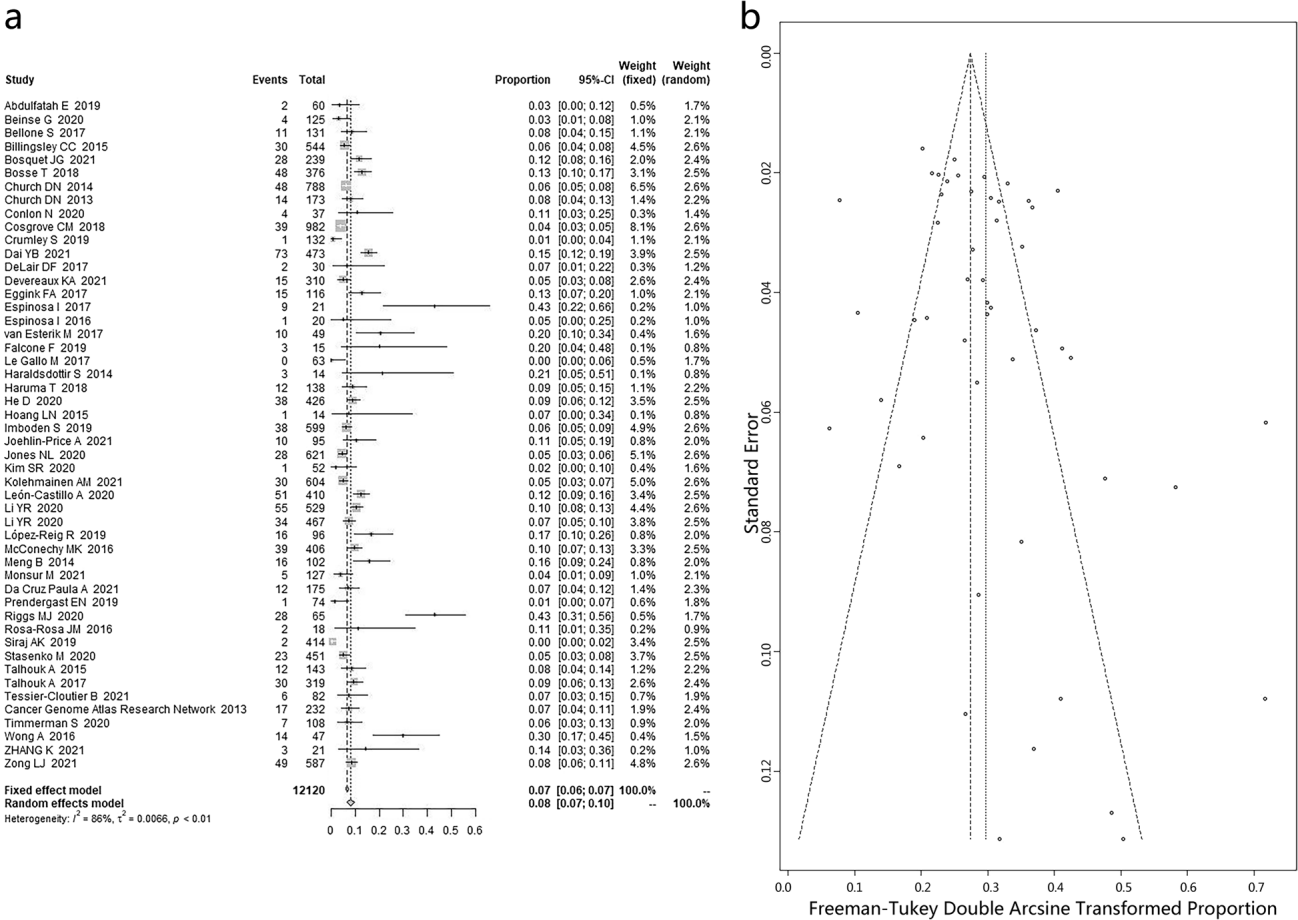
**a** Forest plot and **b** funnel plot for the pooled frequency of POLE-mutated/ultramutated (POLEmut) in endometrial carcinoma (EC)

A total of 49 articles containing 12,120 patients were included in the investigation of frequency of POLEmut ECs. The pooled frequency of POLEmut in ECs was 7.95% (95% CI: 6.52–9.51%) with significant heterogeneity among the studies (*I*^*2*^ = 86.3, 95% CI: 82.7–89.1%, *P* < 0.0001) (Fig. 2a). Furthermore, no publication bias was defined via Egger’s tests (z = 1.832, *P* = 0.06695) and funnel plot (Fig. 2b) in the pooled analysis.

#### Subgroup analyses

We explored subgroup analyses based on histotype, grade, FIGO stage, LVSI, myometrial invasion, lymph node status, clinical risk stratification, and adjuvant therapy. The outcomes of specific subgroup analysis were shown in Table 2. The pooled ORs with 95% CIs were also calculated for POLEmut ECs according to each subgroup variable (Table 1).

**Table 2:** The pooled frequency of POLEmut ECs according to clinicopathology characteristics

Clinicopathological characteristics in EC	Pooled frequency of POLEmut (95% CI), (%)	No. of studies	***I***^**2**^ (95% CI), (%)	***P*** for ***I***^***2***^	Model	Egger’s test
Overall POLEmut	7.95 (6.52–9.51)	50	86.3 (82.7–89.1)	< 0.0001	Random effect	z = 1.832, *P* = 0.06695
EEC	7.95 (6.55–9.46)	32	79.6 (71.8–85.2)	< 0.0001	Random effect	z = 2.5622, *P* = 0.0104
NEEC	4.45 (2.63–6.61)	30	56.0 (33.7–70.8)	0.0001	Random effect	z = 1.018, *P* = 0.3087
Grade 1–2	5.35 (4.16–6.67)	23	57.2 (31.9–73.1)	0.0004	Random effect	z = 1.0836, *P* = 0.2785
Grade 3	10.55 (8.35–12.94)	27	66.6 (50.0–77.7)	< 0.0001	Random effect	z = 0.50043, *P* = 0.6168
FIGO stage I-II	9.15 (7.06–11.46)	29	80.8 (73.2–86.3)	< 0.0001	Random effect	z = 2.7772, *P* = 0.005483
FIGO stage II-IV	3.08 (1.72–4.71)	30	51.9 (26.9–68.3)	0.0006	Random effect	z = 0.66061, *P* = 0.5089
FIGO stage III-IV	2.89 (1.43–4.67)	28	39.4 (4.6–61.6)	0.0180	Random effect	z = 0.25724, *P* = 0.797
LVSI absent	6.96 (5.32–8.77)	17	68.3 (47.6–80.8)	< 0.0001	Random effect	z = 1.7728, *P* = 0.07626
LVSI present	6.40 (3.82–9.48)	17	75.1 (60.0–84.5)	< 0.0001	Random effect	z = 0.24716, *P* = 0.8048
Myometrial invasion≥50%	4.78 (3.47–6.28)	11	39.6 (0.0–70.3)	0.0846	Random effect	z = 0.70065, *P* = 0.4835
Myometrial invasion< 50%	6.85 (5.04–8.89)	11	65.5 (34.5–81.8)	0.0013	Random effect	z = 0.93704, *P* = 0.3487
Lymph node status absent	9.46 (7.77–11.28)	7	0.0 (0.0–45.4)	0.7823	Fixed effect	z = −0.75094, *P* = 0.4527
Lymph node status present	4.97 (0.55–12.07)	7	66.0 (23.9–84.8)	0.0072	Random effect	z = − 0.30722, *P* = 0.7587
Risk stratification-low	5.87 (3.81–8.30)	5	0.0 (0.0–0.0)	0.9660	Fixed effect	z = 0, *P* = 1
Risk stratification-intermediate	7.18 (1.07–16.78)	5	69.4 (21.5–88.0)	0.0110	Random effect	z = 0, *P* = 1
Risk stratification-high	8.87 (6.07–12.09)	7	52.1 (0.0–79.6)	0.0512	Random effect	z = −0.15019, *P* = 0.8806
With adjuvant therapy	9.00 (6.78–11.46)	15	60.5 (30.6–77.6)	0.0012	Random effect	z = 0.14846, *P* = 0.8820
Without adjuvant therapy	6.27 (4.11–8.75)	14	47.0 (1.4–71.5)	0.0266	Random effect	z = 0.4927, *P* = 0.6222

*Abbreviations*: *EC* Endometrial Carcinoma, *POLE* Polymerase ɛ, *POLEmut* POLE-Mutated/Ultramutated, *EEC *Endometrioid Endometrial Carcinoma, *NEEC* Nonendometrioid Endometrial Carcinoma, *FIGO* Federation International of Gynecology and Obstetrics, *LVSI* Lymphovascular Space Invasion, *CI* Confidence Interval

**Table:** **Table 3** The pooled OR of POLEmut ECs according to clinicopathology characteristics

Clinicopathological characteristics in EC	Pooled OR (95% CI)	***P*** for pooled OR	No. of studies	***I***^***2***^ (95% CI), (%)	***P*** for ***I***^***2***^	Model	Egger’s test
EEC vs. NEEC	1.35 (0.88–2.08)	0.1719	22	49.6 (17.4–69.2)	0.0047	Random effects	z = 0.98693, *P* = 0.3237
Grade: 1–2 vs. 3	0.51 (0.36–0.73)	0.0002	22	53.5 (24.6–71.3)	0.0016	Random effects	z = −0.14099, *P* = 0.8879
FIGO stage: I-II vs. III-IV	1.91 (1.29–2.83)	0.0013	28	41.4 (8.0–62.7)	0.0125	Random effects	z = 0.19757, *P* = 0.8434
LVSI: present vs. absent	0.98 (0.77–1.25)	0.8644	17	15.4 (0.0–51.8)	0.2727	Fixed effect	z = −1.6477, *P* = 0.09941
Myometrial invasion: ≥50% vs. < 50%	0.66 (0.50–0.86)	0.0025	10	0.0 (0.0–42.7)	0.7489	Fixed effect	z = −0.98387, *P* = 0.3252
Lymph node status: present vs. absent	1.01 (0.65–1.57)	0.9641	7	23.0 (0.0–65.8)	0.2537	Fixed effect	z = −1.0513, *P* = 0.2931
Clinical risk stratification: high vs. low	1.21 (0.73–2.01)	0.4678	5	0.0 (0.0–75.4)	0.4966	Fixed effect	z = 0, *P* = 1
Adjuvant therapy: yes vs. no	1.16 (0.88–1.54)	0.2939	14	0.0 (0.0–41.7)	0.6918	Fixed effect	z = −0.27372, *P* = 0.7843

*Abbreviations*: *EC* Endometrial Carcinoma; *POLE* Polymerase ɛ; *POLEmut* POLE-Mutated/Ultramutated; *EEC* Endometrioid Endometrial Carcinoma; *NEEC* Nonendometrioid Endometrial Carcinoma; *FIGO* Federation International of Gynecology and Obstetrics; *LVSI* Lymphovascular Space Invasion; *CI* Confidence Interval; *OR* Odds Ratio; vs. Versus

Subgroup analysis was performed based on histotype. A total of 8412 patients with EEC from 32 cohorts were obtained for the meta-analysis. The pooled frequency of POLEmut in EECs was 7.95% (95% CI: 6.55–9.46%) with significant heterogeneity (*I*^*2*^ = 79.6, 95% CI: 71.8–85.2%, *P* < 0.0001). There were 1482 patients from 30 cohorts included for the NEEC meta-analysis. The POLEmut frequency in NEECs was 4.45% (95% CI: 2.63–6.61%) with significant heterogeneity (*I*^*2*^ = 56.0, 95% CI: 33.7–70.8%, *P* < 0.0001). The pooled OR of POLEmut EEC vs. NEEC was 1.35 (95% CI: 0.88–2.08, *P* = 0.1719) with heterogeneity (*I*^*2*^ = 49.6, 95% CI: 17.4–69.2%, *P* = 0.0047).

Subgroup analysis was accomplished based on grade. The pooled frequency of POLEmut ECs was 5.35% (95% CI: 4.16–6.67%) in grade 1–2 and 10.55% (95% CI: 8.35–12.94%) in grade 3. The pooled OR of POLEmut ECs with grade 1–2 vs. grade 3 was 0.51 (95% CI: 0.36–0.73, *P* = 0.0002).

Subgroup analysis was executed based on FIGO stage. The pooled frequency of POLEmut ECs was 9.15% (95% CI: 7.06–11.46%) in FIGO stage I-II and 2.89% (95% CI: 1.43–4.67%) in FIGO stage III-IV. The pooled OR of POLEmut ECs with FIGO stage I-II vs. FIGO stage III-IV was 1.91 (95% CI: 1.29–2.83, *P* = 0.0013).

Subgroup analysis was implemented based on LVSI. The pooled frequency of POLEmut ECs was 6.40% (95% CI: 3.82–9.48%) in LVSI present and 6.96% (95% CI: 5.32–8.77%) in LVSI absent.

Subgroup analysis was carried out based on myometrial invasion. The pooled frequency of POLEmut ECs was 4.78% (95% CI: 3.47–6.28%) in myometrial invasion ≥50 and 6.85% (95% CI: 5.04–8.89%) in myometrial invasion < 50%. The pooled OR of POLEmut ECs with myometrial invasion ≥50% vs. myometrial invasion < 50% was 0.66 (95% CI: 0.50–0.86, *P* = 0.0025).

Subgroup analysis was performed based on lymph node status. The pooled frequency of POLEmut ECs was 4.97% (95% CI: 0.55–12.07%) in lymph node status present and 9.46% (95% CI: 7.77–11.28%) in lymph node status absent.

Subgroup analysis was accomplished based on clinical risk stratification. The pooled frequency of POLEmut ECs was 5.87% (95% CI: 3.81–8.30%) in low-risk stratification, 7.18% (95%CI: 1.07–16.78%) in intermediate-risk stratification, and 8.87% (95% CI: 6.07–12.09%) in high-risk stratification.

Subgroup analysis was conducted based on with or without adjuvant therapy. The pooled frequency of POLEmut ECs was 9.00% (95% CI: 6.78–11.46%) with adjuvant therapy, and 6.27% (95% CI: 4.11–8.75%) without adjuvant therapy.

#### The frequency of other molecular subtypes (MSI and p53abn) in ECs

The pooled frequency of MSI in ECs was 27.23% (95% CI: 23.66–30.95%) (Fig. S1a) with significant heterogeneity among studies (*I*^*2*^ = 91.1, 95% CI: 88.6–93.0%, *P* < 0.0001) (Table S3); the pooled frequency of p53abn in ECs was 23.47% (95% CI: 19.70–27.46%) (Fig. S1b) with significant heterogeneity among studies (*I*^*2*^ = 90.8, 95% CI: 88.0–93.0%, *P* < 0.0001) (Table S3). No publication bias was calculated via Egger’s tests (Table S3) and funnel plot (Fig. S1c, d) in the pooled analyses.

#### Survival analyses

**Fig. 3:**
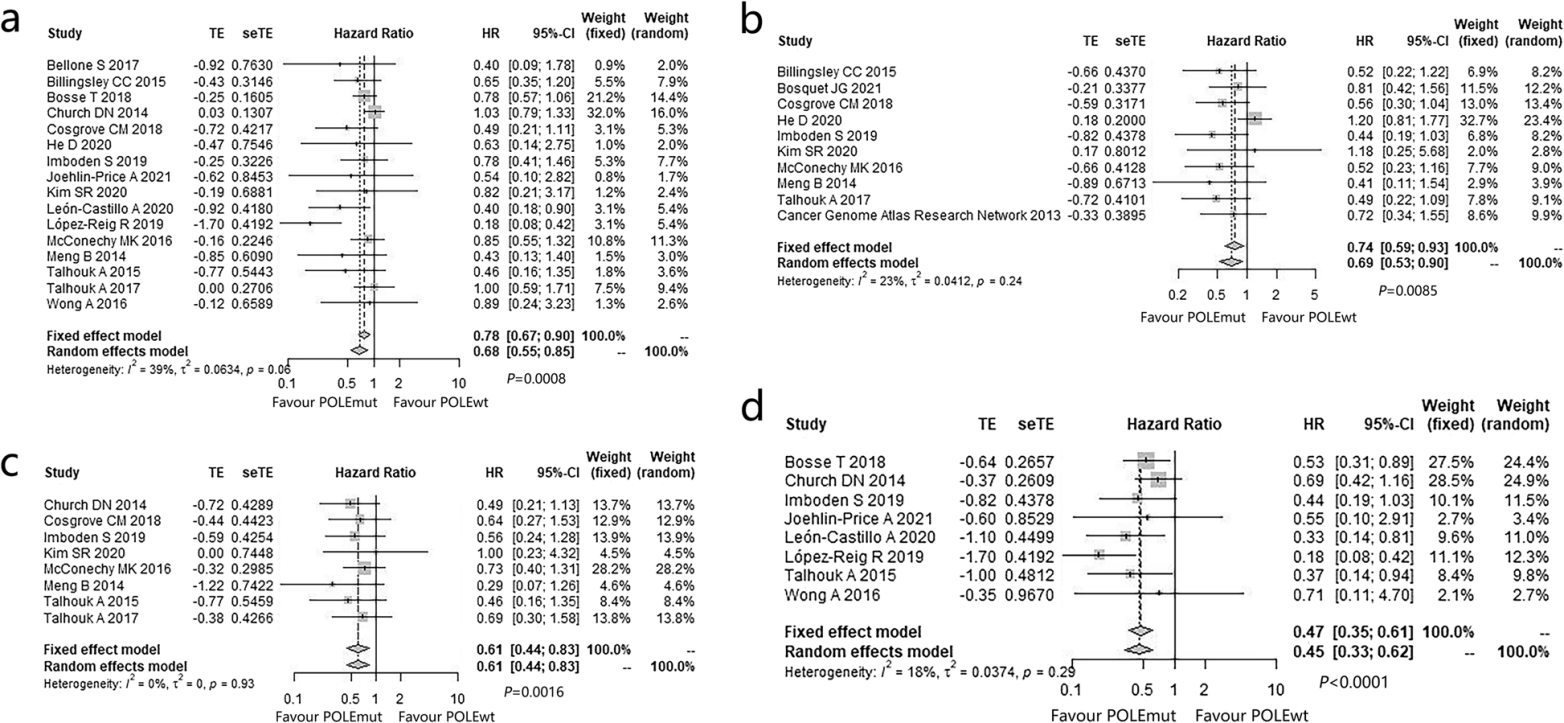
Forest plot of the meta-analysis estimating the hazard ratio (HR) with 95% confidence interval (CI) of **a** overall survival (OS), **b** progression free survival (PFS), **c** disease specific survival (DSS), and **d** relapse free survival (RFS) for POLEmut compared with POLE-wild-type (POLEwt) EC patients

Survival analyses were displayed by pooled HRs with 95% CIs for OS, PFS, DSS, and RFS. Of all the adopted studies, 16 cohorts contained data for OS, 10 for PFS, 8 for DSS, and 8 for RFS. The pooled HRs of POLEmut vs. POLE-wild-type (POLEwt) ECs were 0.68 (95% CI: 0.55–0.85, *P* = 0.0008) for OS (Fig. 3a), 0.74 (95% CI: 0.59–0.93, *P* = 0.0085) for PFS (Fig. 3b), 0.61 (95% CI: 0.44–0.83, *P* = 0.0016) for DSS (Fig. 3c), and 0.47 (95% CI: 0.35–0.61, *P* < 0.0001) for RFS (Fig. 3d). These results indicated benefit survival and favorable prognosis in POLEmut EC patients. No publication bias was calculated via funnel plot (Fig. S2) in the pooled analyses.

Additionally, univariable and multivariable analyses were pooled to test the associations among the four molecular subtypes (POLEmut, MSI, p53wt and p53abn) with clinical outcomes (OS, PFS, DSS and RFS) in ECs (Table 3). The results revealed that the clinical outcomes of POLEmut group were the best, but those of p53abn group were the worst, while those of MSI group and p53wt group were medium.

**Table 3:** The pooled HRs of OS, PFS, DSS, RFS for four molecular subtypes at univariable and multivariable analyses

		Univariable analyses					Multivariable analyses				
		pooled HR (95%CI)	*P* of HR	*I*^*2*^ (95%CI)	*P* of *I*^*2*^	Number of studies	pooled HR (95%CI)	*P* of HR	*I*^*2*^ (95%CI)	*P* of *I*^*2*^	Number of studies
OS	POLEmut vs. p53wt	0.69 (0.55–0.87)	0.0016	0.0% (0.0–62.5%)	0.6952	5	0.77 (0.60–0.99)	0.0447	0.0% (0.0–83.4%)	0.4276	4
	MSI vs. p53wt	1.15 (0.97–1.37)	0.1054	59.9% (1.6–83.7%)	0.0288	6	1.08 (0.94–1.25)	0.2620	40.9% (0.0–78.2%)	0.1489	5
	p53mt vs p53 wt	1.40 (1.15–1.71)	0.0007	66.0% (11.3–87.0%)	0.0192	5	1.24 (1.09–1.40)	0.0009	0.0% (0.0–80.0%)	0.5141	4
PFS	POLEmut vs. p53wt	0.66 (0.42–1.04)	0.0743	0.0% (0.0–76.3%)	0.6447	3	0.53 (0.32–0.87)	0.0112	0.00%	0.7927	2
	MSI vs. p53wt	1.29 (0.92–1.82)	0.1421	59.9% (0.0–88.6%)	0.0828	3	1.01 (0.88–1.15)	0.9029	7.90%	0.2973	2
	p53mt vs p53 wt	1.81 (1.24–2.66)	0.0023	79.2% (33.7–93.5%)	0.0082	3	1.23 (1.04–1.46)	0.0186	0.00%	0.8026	2
DSS	POLEmut vs. p53wt	0.81 (0.52–1.26)	0.3392	0.0% (0.0–13.3%)	0.9123	4	0.62 (0.36–1.07)	0.0867	0.0% (0.0–21.5%)	0.8759	3
	MSI vs. p53wt	1.04 (0.69–1.57)	0.8534	74.8% (30.1–90.9%)	0.0076	4	1.02 (0.84–1.24)	0.8258	0.0% (0.0–47.6%)	0.8199	3
	p53mt vs p53 wt	1.77 (1.51–2.09)	< 0.0001	16.5% (0.0–87.2%)	0.3087	4	1.34 (1.09–1.66)	0.0051	0.0% (0.0–62.5%)	0.7579	3
RFS	POLEmut vs. p53wt	0.46 (0.29–0.74)	0.0015	0.00%	0.9695	2	0.50 (0.31–0.80)	0.0038	0.00%	0.5722	2
	MSI vs. p53wt	0.92 (0.81–1.06)	0.2449	0.0% (0.0–86.9%)	0.4521	3	0.89 (0.78–1.02)	0.1035	34.6% (0.0–78.7%)	0.2167	3
	p53mt vs p53 wt	1.47 (1.14–1.89)	0.0030	50.9% (0.0–85.8%)	0.1306	3	1.35 (1.04–1.74)	0.0221	49.0% (0.0–85.2%)	0.141	3

*Abbreviations*: *POLEmut* POLE-Mutated/Ultramutated; *MSI* Microsatellite-Instable/Hypermutated; *p53abn* p53-Abnormal/Mutated; *p53wt*: p53-Wild-Type; *OS* Overall Survival; *PFS* Progression Free Survival; *DSS* Disease Specific Survival; *RFS* Relapse Free Survival; *CI* Confidence Interval; *HR* Hazard Ratio

#### Assessment of study quality

All the studies were highly qualified (quality assessment of 49 included articles is summarized in Table S4) with relatively satisfying results for bias risk assessment.

## Discussion

Worldwide, EC is one of the most common cancers of women with survival rate not improving. TCGA research network firstly identified the molecular cohort of POLEmut EC that features a favorable prognostic potential, despite with bad clinicopathological parameters [22]. Accumulating studies were conducted on the POLEmut, but the frequency and prognostic value of POLEmut in EC patients were variable among previous researches [3, 23–25]. Therefore, this study aimed to estimate the frequency and clinicopathological characteristics of POLEmut and the overall effect on prognosis of EC patients.

Our study revealed that 7.95% (95% CI: 6.52–9.51%) of EC patients harbored POLEmut. The results exhibited that there were no significant differences in histotype (EEC vs. NEEC) of POLEmut ECs; and no significant relations were observed between POLEmut and LVSI, lymph node status, clinical risk stratification, or adjuvant therapy. However, it should be noted that histotype and LVSI are features that generally subjective with interobserver variability and may not be reproducible between series [6, 26]. The vast majority of it presented higher expression at earlier stage and less myometrial invasion, both of which were “traditional” identified as an important marker of low-risk stratification; meanwhile, the POLEmut ECs presented at the highest grade (grade 3), which were generally considered to be associated with a higher risk of recurrence and death [27].

Studies have confirmed that POLEmut ECs had better clinical outcomes with survival analysis, even those at high grade [28–30]. Paradoxically, some investigators advocated that superior survival was not found in POLEmut ECs [19, 20]. Based on our study, EC patients with POLEmut possessed better clinical survivals (including OS, PFS, DSS, and RFS) than those with POLEwt. Additionally, according to both pooled univariable and multivariable analyses, the POLEmut cohort showed the best clinical prognosis among the four molecular subtypes, with a death risk of any cause lower than that of other three molecular subtypes, and a risk of recurrent/progressive disease lower; while the p53abn group, as expected, showed the worst prognosis. The reason why POLEmut correlates favorable outcomes in the patients remains unclear. Meng et al. [31] had speculated that this might due to the high mutation burden and the increase in base substitution; Howitt et al. [32] showed that POLEmut ECs were associated with high neoantigens and elevated CD8+ tumor infiltrating lymphocytes, which was counterbalanced by overexpression of program death-ligand. POLE proofreading mutations might elicit an anti-tumor response [33].

There is now an emerging link between high mutation burden in tumors and improved prognosis in cancer patients. Indeed, POLEmut tumors have been shown to feature higher immune infiltrations and programmed death-1 (PD-1) and programmed death-ligand 1 (PD-L1) expression [34], which may offset the survival risk caused by higher tumor grades in ultramutated POLE and thus generate a favorable prognosis. Consequently, POLEmut in EC patients was a promising terapeutical target [35].

Talhouk et al. [4] found that half of POLEmut ECs were identified as with “high risk” based on stage, histology, and grade. It is clear that there may be both over-treatment and under-treatment of women based solely on application of the previous risk-assessment tool. In 2020, the European Society of Gynaecological Oncology (ESGO)/ European Society for Radiotherapy and Oncology (ESTRO)/ European Society of Pathology (ESP) published their joint guidelines for the management of EC, for the first time incorporating the TCGA findings [including groups of POLEmut, MMRd, p53abn and NSMP (surrogate of the copy number low/endometrioid group)] to assess the prognosis of EC in association with classic and distinct clinicopathologic prognostic factors (such as stage, grade, histotype, myometrial invasion or LVSI) in the risk stratification of EC [36]. However, several points remain to be clarified, as the prognostic value of the TCGA molecular group may vary among diverse histotypes of EC [37]. It has been recorded that POLEmut served as the molecular signature least affected by other prognostic clinicopathological factors [38]. Furthermore, based on our study, there was no significant difference in frequency of POLEmut between EC patients with and without adjuvant therapy. For this reason, the clinical practice that many of the patients currently undergo adjuvant treatment may constitute an overtreatment. It is reasonable to identify POLEmut status at the moment of diagnosis and to mete out less intensive treatment for EC patients with POLEmut.

It remains obscure whether the favorable clinical outcomes observed in patients with POLEmut ECs were independent of the receipt of adjuvant therapy. Furthermore, other molecular factors and clinicopathological might have an independent prognostic value in the context of the TCGA classification [38], such as the LVSI [39]. Therefore, novel initiatives stratifying ECs for clinical trials according to molecular subtype are recommended, since they will provide a key step toward precision medicine for ECs.

## Limitations

This study came across three drawbacks: firstly, there were only 8 prospective studies despite containing 49 articles involving 12,120 patients, for analyzing the clinicopathological characteristics of POLEmut ECs and prognostic value of POLE status; secondly, bias might exist to some extent for excluding relevant studies published in non-English language; the last was that the heterogeneity of included studies was high to some degree.

## Conclusions

The POLEmut emergered higher expression in ECs with grade 3, FIGO stage I-II, and myometrial invasion< 50%; it might serve as a highly favorable prognostic marker in EC; the clinical outcomes of POLEmut group were the best one among the four molecular subtypes.

## Supplementary Information

**Additional file 1: Table S1.** PRISMA

**Additional file 2: Table S2.** The list of the included studies.

**Additional file 3: Figure S1.** Forest plot for the pooled frequency of (a) microsatellite-instable(MSI)/hypermutated and (b) p53-abnormal/mutated (p53abn) in endometrial carcinoma (EC); funnel plot for the pooled frequency of (c) MSI and (d) p53abn in EC.

**Additional file 4: Table S3.** The proportion of MSI and p53abn molecular subtypes in ECs.

**Additional file 5: Figure S2.** Funnel plot of (a) overall survival (OS), (b) progression-free survival (PFS), (c) disease specific survival (DSS), and (d) relapse free survival (RFS) for POLEmut compared with POLEwt EC patients.

**Additional file 6: Table S4.** The Newcastle-Ottawa scale for quality assessment of the studies.

## Data Availability

The datasets generated during and/or analyzed during the current study are available from the corresponding author on reasonable request.

## Abbreviations

EC: Endometrial Carcinoma
POLE: Polymerase ɛ
POLEmut: POLE-Mutated/Ultramutated
MSI: Microsatellite-Instable/Hypermutated
p53abn: p53-Abnormal/Mutated
p53wt: p53-Wild-Type
ICIs: Immune Checkpoint Inhibitor
NA: Not Available
EEC: Endometrioid Endometrial Carcinoma
NEEC: Nonendometrioid Endometrial Carcinoma
OS: Overall Survival
PFS: Progression Free Survival
DSS: Disease Specific Survival
RFS: Relapse Free Survival
FIGO: Federation International of Gynecology and Obstetrics
ProMisE: Proactive Molecular Risk Classifier for Endometrial Cancer
LVSI: Lymphovascular Space Invasion
CI: Confidence Interval
HR: Hazard Ratio
OR: Odd Ratio
NOS: Newcastle-Ottawa Scale
ESGO: Gynaecological Oncology
ESTRO: European Society for Radiotherapy and Oncology
ESP: European Society of Pathology
