# Supplementary material for: The clinicopathological characteristics of POLE-mutated/ultramutated endometrial carcinoma and prognostic value of POLE status: a meta-analysis based on 49 articles incorporating 12,120 patients
Source: BMC Cancer. 2022 Nov 10;22:1157. doi: 10.1186/s12885-022-10267-2 (PMC9647950; doi:10.1186/s12885-022-10267-2)
Supplement: Supplementary file 2 — Additional file 2: Table S2. The list of the included studies. [file 12885_2022_10267_MOESM2_ESM.docx]

**Table S2 The list of the included studies**

| **The First Author** | **Title** | **Journal** | **Year** | **Volume** | **Issue** | **Page** |
| --- | --- | --- | --- | --- | --- | --- |
| Abdulfatah E | Molecular classification of endometrial carcinoma applied to endometrial biopsy specimens: Towards early personalized patient management. | Gynecol Oncol | 2019 | 154 | 3 | 467-474 |
| Beinse G | Identification of TP53 mutated group using a molecular and immunohistochemical classification of endometrial carcinoma to improve prognostic evaluation for adjuvant treatments. | Int J Gynecol Cancer | 2020 | 30 | 5 | 640-647 |
| Bellone S | Polymerase ε (POLE) ultra-mutation in uterine tumors correlates with T lymphocyte infiltration and increased resistance to platinum-based chemotherapy in vitro. | Gynecol Oncol | 2017 | 144 | 1 | 146-152 |
| Billingsley CC | Polymerase ɛ (POLE) mutations in endometrial cancer: clinical outcomes and implications for Lynch syndrome testing. | Cancer | 2015 | 121 | 3 | 386-94 |
| Bosquet JG | Association of a novel endometrial cancer biomarker panel with prognostic risk, platinum insensitivity, and targetable therapeutic options. | PLoS One | 2021 | 16 | 1 | e0245664 |
| Bosse T | Molecular Classification of Grade 3 Endometrioid Endometrial Cancers Identifies Distinct Prognostic Subgroups. | Am J Surg Pathol | 2018 | 42 | 5 | 561-568 |
| Church DN | Prognostic significance of POLE proofreading mutations in endometrial cancer. | J Natl Cancer Inst | 2014 | 107 | 1 | 402 |
| Church DN | DNA polymerase ε and δ exonuclease domain mutations in endometrial cancer. | Hum Mol Genet | 2013 | 22 | 14 | 336238 |
| Conlon N | Endometrial Carcinomas with a "Serous" Component in Young Women Are Enriched for DNA Mismatch Repair Deficiency, Lynch Syndrome, and POLE Exonuclease Domain Mutations. | Am J Surg Pathol | 2020 | 44 | 5 | 641-648 |
| Cosgrove CM | An NRG Oncology/GOG study of molecular classification for risk prediction in endometrioid endometrial cancer. | Gynecol Oncol | 2018 | 148 | 1 | 174-180 |
| Crumley S | Identification of a subset of microsatellite-stable endometrial carcinoma with high PD-L1 and CD8+ lymphocytes. | Mod Pathol | 2019 | 32 | 3 | 396-404 |
| Dai YB | Tumor Molecular Features Predict Endometrial Cancer Patients' Survival After Open or Minimally Invasive Surgeries. | Front Oncol | 2021 | 11 |  | 634857 |
| DeLair DF | The genetic landscape of endometrial clear cell carcinomas. | J Pathol | 2017 | 243 | 2 | 230-241 |
| Devereaux KA | Prospective molecular classification of endometrial carcinomas: institutional implementation, practice, and clinical experience. | Mod Pathol | 2021 |  |  |  |
| Eggink FA | Immunological profiling of molecularly classified high-risk endometrial cancers identifies POLE-mutant and microsatellite unstable carcinomas as candidates for checkpoint inhibition. | Oncoimmunology | 2017 | 6 | 2 | e1264565 |
| Espinosa I | Undifferentiated and Dedifferentiated Endometrial Carcinomas With POLE Exonuclease Domain Mutations Have a Favorable Prognosis. | Am J Surg Pathol | 2017 | 41 | 8 | 1121-1128 |
| Espinosa I | Mixed and Ambiguous Endometrial Carcinomas: A Heterogenous Group of Tumors With Different Clinicopathologic and Molecular Genetic Features. | Am J Surg Pathol | 2016 | 40 | 7 | 972-81 |
| van Esterik M | Limited impact of intratumour heterogeneity on molecular risk assignment in endometrial cancer. | Oncotarget | 2017 | 8 | 15 | 25542-25551 |
| Falcone F | Application of the Proactive Molecular Risk Classifier for Endometrial Cancer (ProMisE) to patients conservatively treated: Outcomes from an institutional series. | Eur J Obstet Gynecol Reprod Biol | 2019 | 240 |  | 220-225 |
| Le Gallo M | Somatic mutation profiles of clear cell endometrial tumors revealed by whole exome and targeted gene sequencing. | Cancer | 2017 | 123 | 17 | 3261-3268 |
| Haraldsdottir S | Colon and endometrial cancers with mismatch repair deficiency can arise from somatic, rather than germline, mutations. | Gastroenterology | 2014 | 147 | 6 | 1308-1316 |
| Haruma T | Clinical impact of endometrial cancer stratified by genetic mutational profiles, POLE mutation, and microsatellite instability. | PLoS One | 2018 | 13 | 4 | e0195655 |
| He D | POLE mutation combined with microcystic, elongated and fragmented (MELF) pattern invasion in endometrial carcinomas might be associated with poor survival in Chinese women. | Gynecol Oncol | 2020 | 159 | 1 | 36-42 |
| Hoang LN | Targeted mutation analysis of endometrial clear cell carcinoma. | Histopathology | 2015 | 66 | 5 | 664-74 |
| Imboden S | Phenotype of POLE-mutated endometrial cancer. | PLoS One | 2019 | 14 | 3 | e0214318 |
| Joehlin-Price A | Molecularly Classified Uterine FIGO Grade 3 Endometrioid Carcinomas Show Distinctive Clinical Outcomes But Overlapping Morphologic Features. | Am J Surg Pathol | 2021 | 45 | 3 | 421-429 |
| Jones NL | Immune checkpoint expression, microsatellite instability, and mutational burden: Identifying immune biomarker phenotypes in uterine cancer. | Gynecol Oncol | 2020 | 156 | 2 | 393-399 |
| Kim SR | Molecular subtypes of clear cell carcinoma of the endometrium: Opportunities for prognostic and predictive stratification. | Gynecol Oncol | 2020 | 158 | 1 | 3-11 |
| Kolehmainen AM | Molecular characterization in the prediction of disease extent in endometrial carcinoma. | Eur J Obstet Gynecol Reprod Biol | 2021 | 256 |  | 478-483 |
| León-Castillo A | Molecular Classification of the PORTEC-3 Trial for High-Risk Endometrial Cancer: Impact on Prognosis and Benefit From Adjuvant Therapy. | J Clin Oncol | 2020 | 38 | 29 | 3388-3397 |
| Li YR | POLE Mutation Characteristics in a Chinese Cohort with Endometrial Carcinoma. | Onco Targets Ther | 2020 | 13 |  | 7305-7316 |
| López-Reig R | Prognostic classification of endometrial cancer using a molecular approach based on a twelve-gene NGS panel. | Sci Rep | 2019 | 9 | 1 | 18093 |
| McConechy MK | Endometrial Carcinomas with POLE Exonuclease Domain Mutations Have a Favorable Prognosis. | Clin Cancer Res | 2016 | 22 | 12 | 2865-73 |
| Meng B | POLE exonuclease domain mutation predicts long progression-free survival in grade 3 endometrioid carcinoma of the endometrium. | Gynecol Oncol | 2014 | 134 | 1 | 15-9 |
| Monsur M | Endometrial cancer with a POLE mutation progresses frequently through the type I pathway despite its high-grade endometrioid morphology: a cohort study at a single institution in Japan. | Med Mol Morphol | 2021 | 54 | 2 | 133-145 |
| Da Cruz Paula A | Genetic and molecular subtype heterogeneity in newly diagnosed early- and advanced-stage endometrial cancer. | Gynecol Oncol | 2021 | 161 | 2 | 535-544 |
| Prendergast EN | Comprehensive genomic profiling of recurrent endometrial cancer: Implications for selection of systemic therapy. | Gynecol Oncol | 2019 | 154 | 3 | 461-466 |
| Riggs MJ | DACH1 mutation frequency in endometrial cancer is associated with high tumor mutation burden. | PLoS One | 2020 | 15 | 12 | e0244558 |
| Rosa-Rosa JM | Molecular genetic heterogeneity in undifferentiated endometrial carcinomas. | Mod Pathol | 2016 | 29 | 11 | 1390-1398 |
| Siraj AK | Germline POLE and POLD1 proofreading domain mutations in endometrial carcinoma from Middle Eastern region. | Cancer Cell Int | 2019 | 19 |  | 334 |
| Stasenko M | Clinical outcomes of patients with POLE mutated endometrioid endometrial cancer. | Gynecol Oncol | 2020 | 156 | 1 | 194-202 |
| Talhouk A | A clinically applicable molecular-based classification for endometrial cancers. | Br J Cancer | 2015 | 113 | 2 | 299-310 |
| Talhouk A | Confirmation of ProMisE: A simple, genomics-based clinical classifier for endometrial cancer. | Cancer | 2017 | 123 | 5 | 802-813 |
| Tessier-Cloutier B | SWI/SNF-deficiency defines highly aggressive undifferentiated endometrial carcinoma. | J Pathol Clin Res | 2021 | 7 | 2 | 144-153 |
| Cancer Genome Atlas Research Network | Integrated genomic characterization of endometrial carcinoma. | Nature | 2013 | 497 | 7447 | 67-73 |
| Timmerman S | Analysis of 108 patients with endometrial carcinoma using the PROMISE classification and additional genetic analyses for MMR-D. | Gynecol Oncol | 2020 | 157 | 1 | 245-251 |
| Wong A | Mutation spectrum of POLE and POLD1 mutations in South East Asian women presenting with grade 3 endometrioid endometrial carcinomas. | Gynecol Oncol | 2016 | 141 | 1 | 113-20 |
| ZHANG K | Clinicopathological significance of multiple molecular features in undifferentiated and dedifferentiated endometrial carcinomas. | Pathology | 2021 | 53 | 2 | 179-186 |
| Zong LJ | PD-L1 expression in tumor cells is associated with a favorable prognosis in patients with high-risk endometrial cancer. | Gynecol Oncol | 2021 | 162 | 3 | 631-637 |
