# Supplementary material for: The clinicopathological characteristics of POLE-mutated/ultramutated endometrial carcinoma and prognostic value of POLE status: a meta-analysis based on 49 articles incorporating 12,120 patients
Source: BMC Cancer. 2022 Nov 10;22:1157. doi: 10.1186/s12885-022-10267-2 (PMC9647950; doi:10.1186/s12885-022-10267-2)
Supplement: Supplementary file 3 — Additional file 3: Figure S1. Forest plot for the pooled frequency of (a) microsatellite-instable(MSI)/hypermutated and (b) p53-abnormal/mutated (p53abn) in endometrial carcinoma (EC); funnel plot for the pooled frequency of (c) MSI and (d) p53abn in EC. [file 12885_2022_10267_MOESM3_ESM.docx]

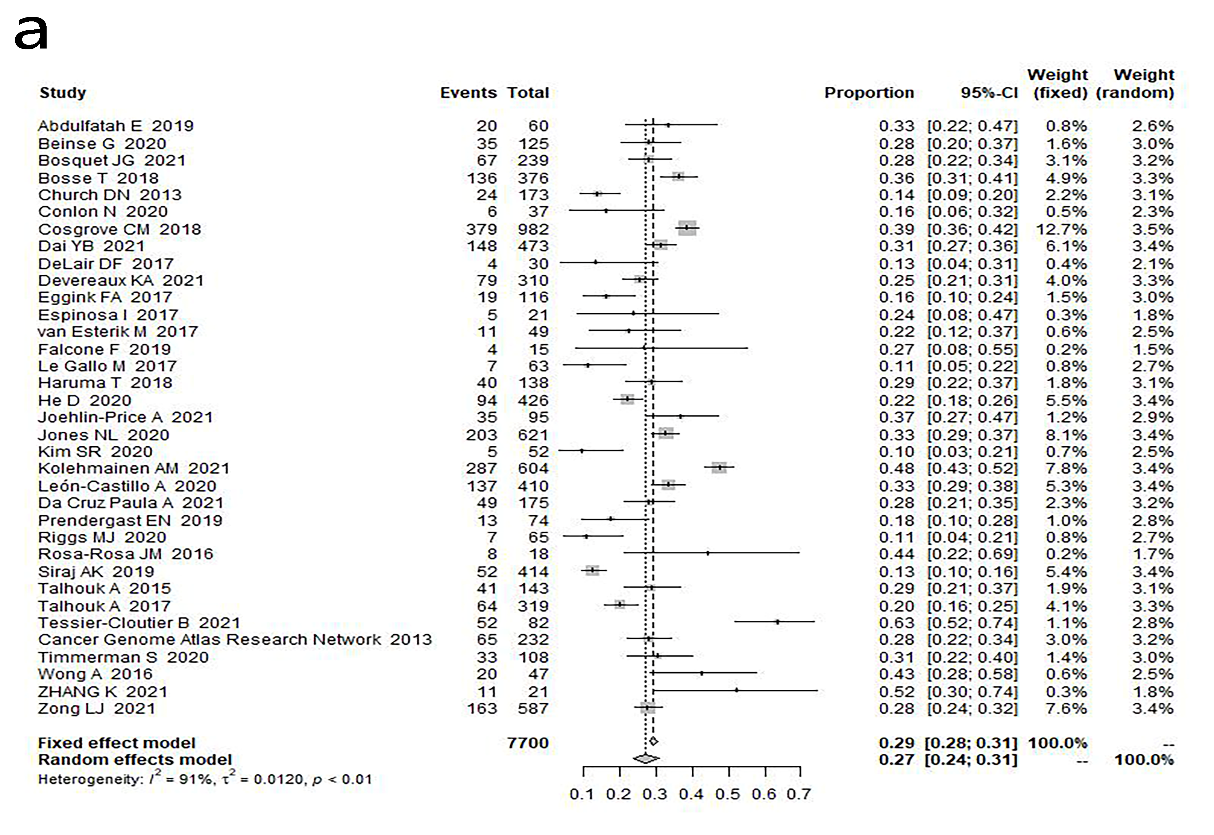


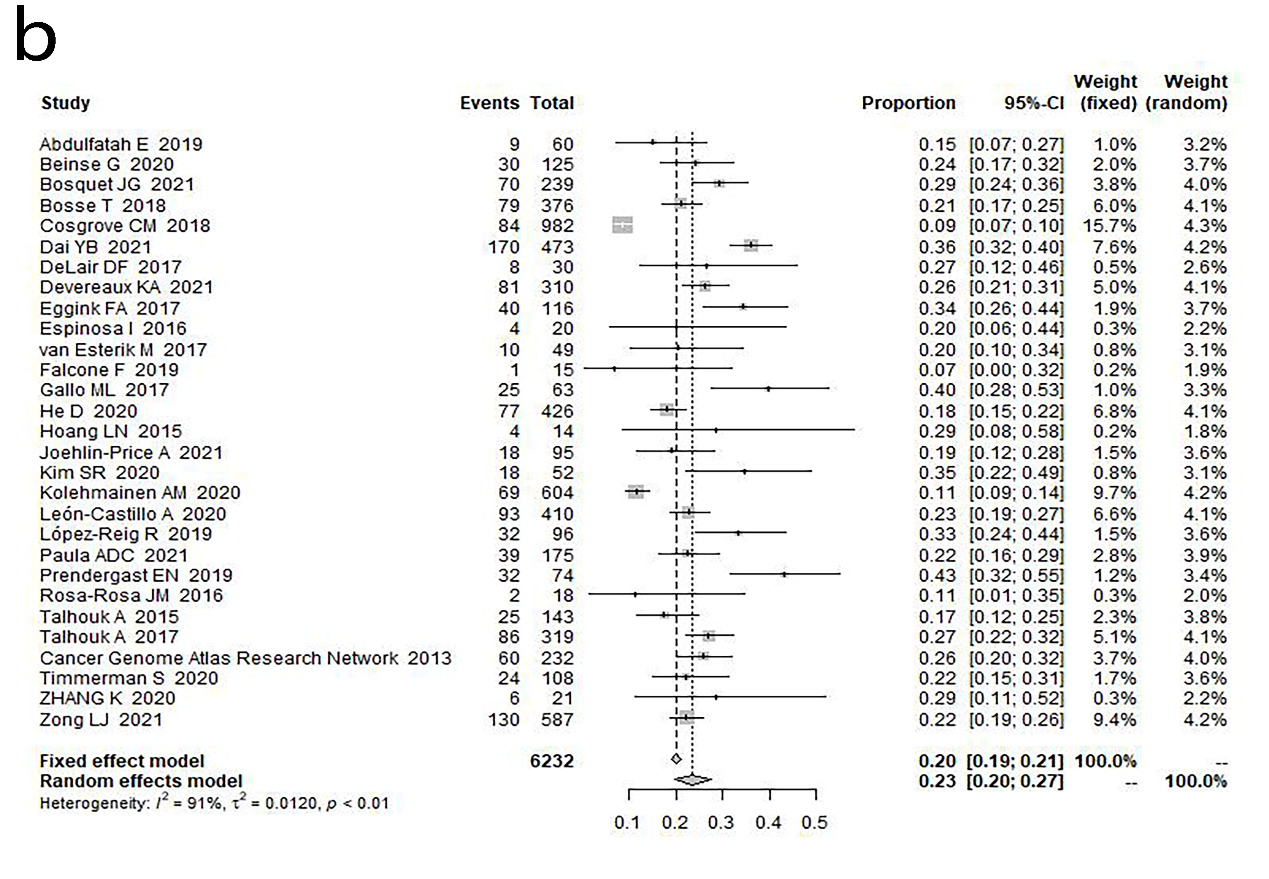

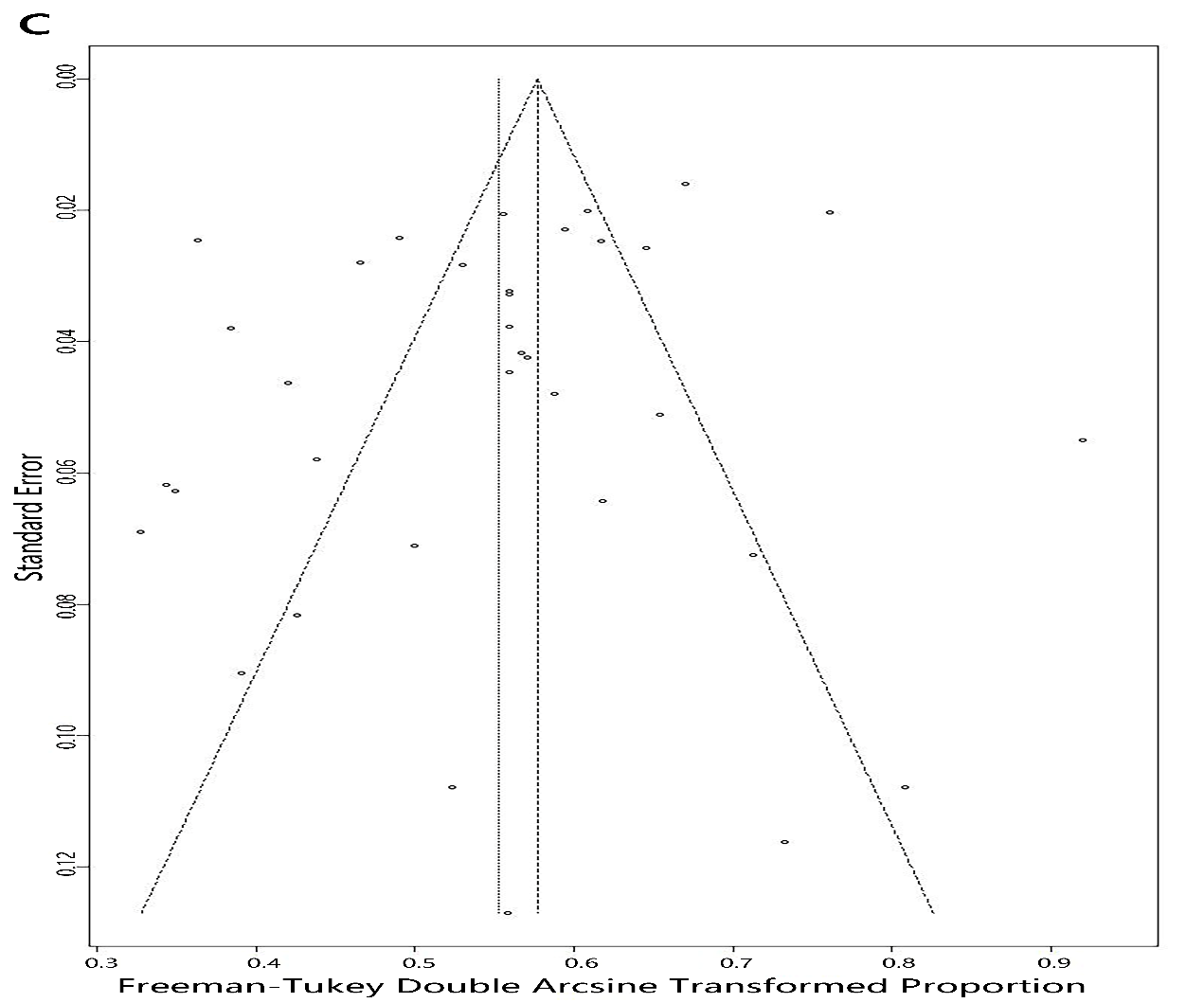


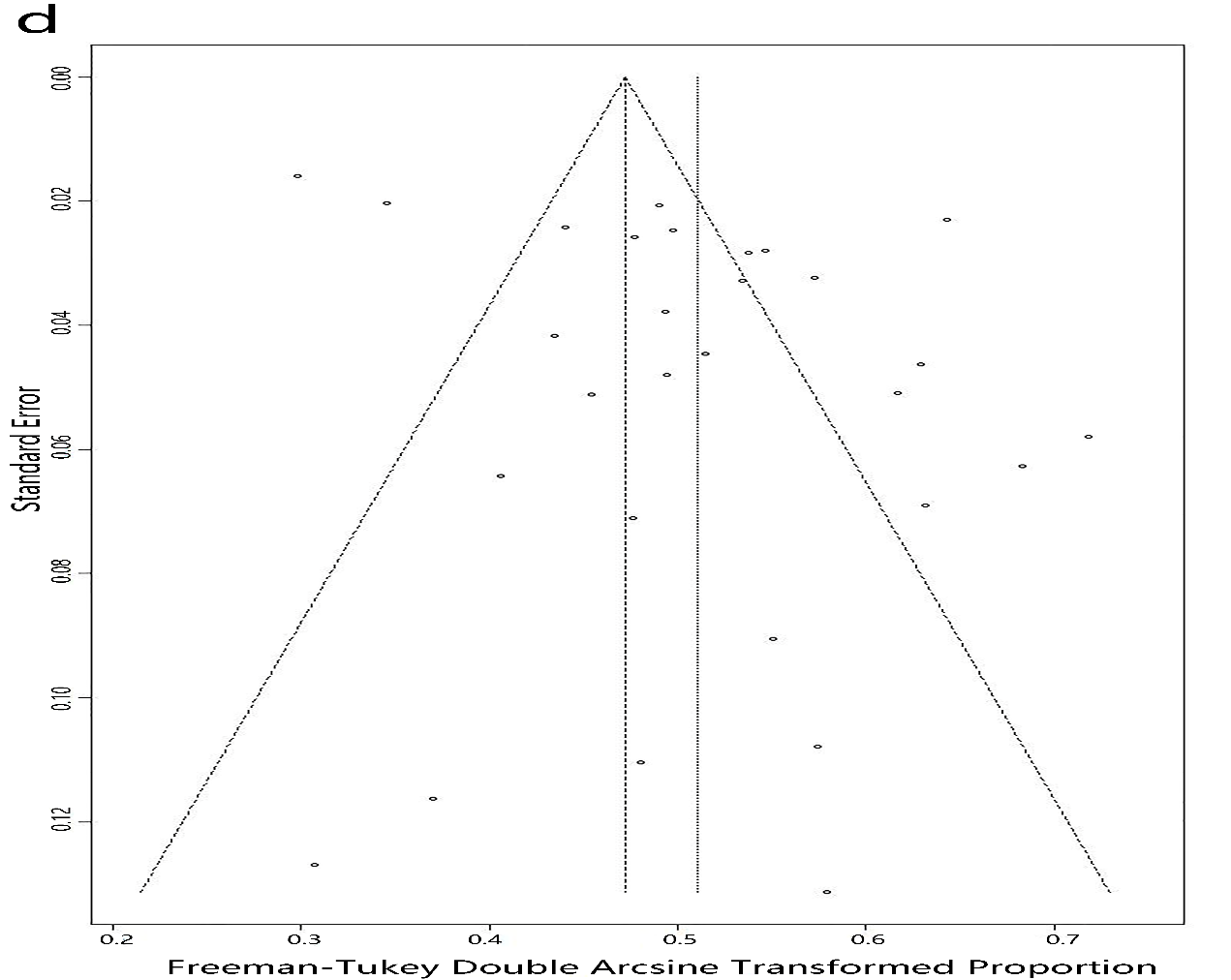


**Fig. S1** Forest plot for the pooled frequency of (a) microsatellite-instable(MSI)/hypermutated and (b) p53-abnormal/mutated (p53abn) in endometrial carcinoma (EC); funnel plot for the pooled frequency of (c) MSI and (d) p53abn in EC.
