# Supplementary material for: The clinicopathological characteristics of POLE-mutated/ultramutated endometrial carcinoma and prognostic value of POLE status: a meta-analysis based on 49 articles incorporating 12,120 patients
Source: BMC Cancer. 2022 Nov 10;22:1157. doi: 10.1186/s12885-022-10267-2 (PMC9647950; doi:10.1186/s12885-022-10267-2)
Supplement: Supplementary file 4 — Additional file 4: Table S3. The proportion of MSI and p53abn molecular subtypes in ECs. [file 12885_2022_10267_MOESM4_ESM.docx]

**Table S3 The proportion of MSI and p53abn molecular subtypes in ECs**

| **Genotype** | **Pooled rate (95% CI), (%)** | **No. of studies** | ***I^2^* (95% CI), (%)** | ***P* value** | **Model** | **Egger’s test** |
| --- | --- | --- | --- | --- | --- | --- |
| MSI | 27.23 (23.66-30.95) | 35 | 91.1 (88.6-93.0) | <0.0001 | Random effect | z = -0.14203, *P* = 0.8871 |
| p53abn | 23.47 (19.70-27.46) | 29 | 90.8 (88.0-93.0) | <0.0001 | Random effect | z = -0.30013, *P* = 0.7641 |

Abbreviations: EC, endometrial carcinoma; MSI, microsatellite-instable/hypermutated; p53abn, p53-abnormal/mutated; CI, confidence interval.
