# Supplementary material for: The clinicopathological characteristics of POLE-mutated/ultramutated endometrial carcinoma and prognostic value of POLE status: a meta-analysis based on 49 articles incorporating 12,120 patients
Source: BMC Cancer. 2022 Nov 10;22:1157. doi: 10.1186/s12885-022-10267-2 (PMC9647950; doi:10.1186/s12885-022-10267-2)
Supplement: Supplementary file 5 — Additional file 5: Figure S2. Funnel plot of (a) overall survival (OS), (b) progression-free survival (PFS), (c) disease specific survival (DSS), and (d) relapse free survival (RFS) for POLEmut compared with POLEwt EC patients. [file 12885_2022_10267_MOESM5_ESM.docx]

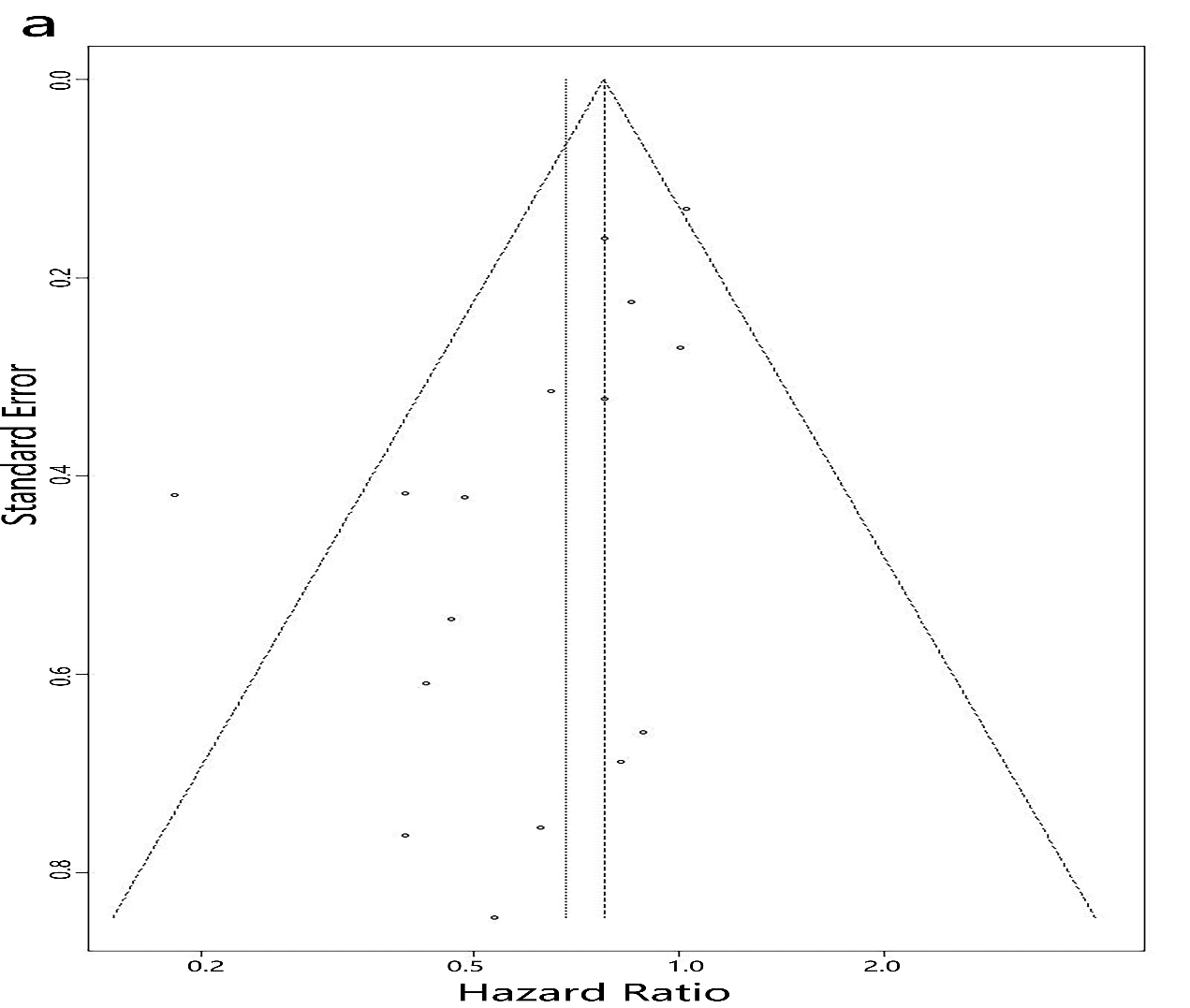


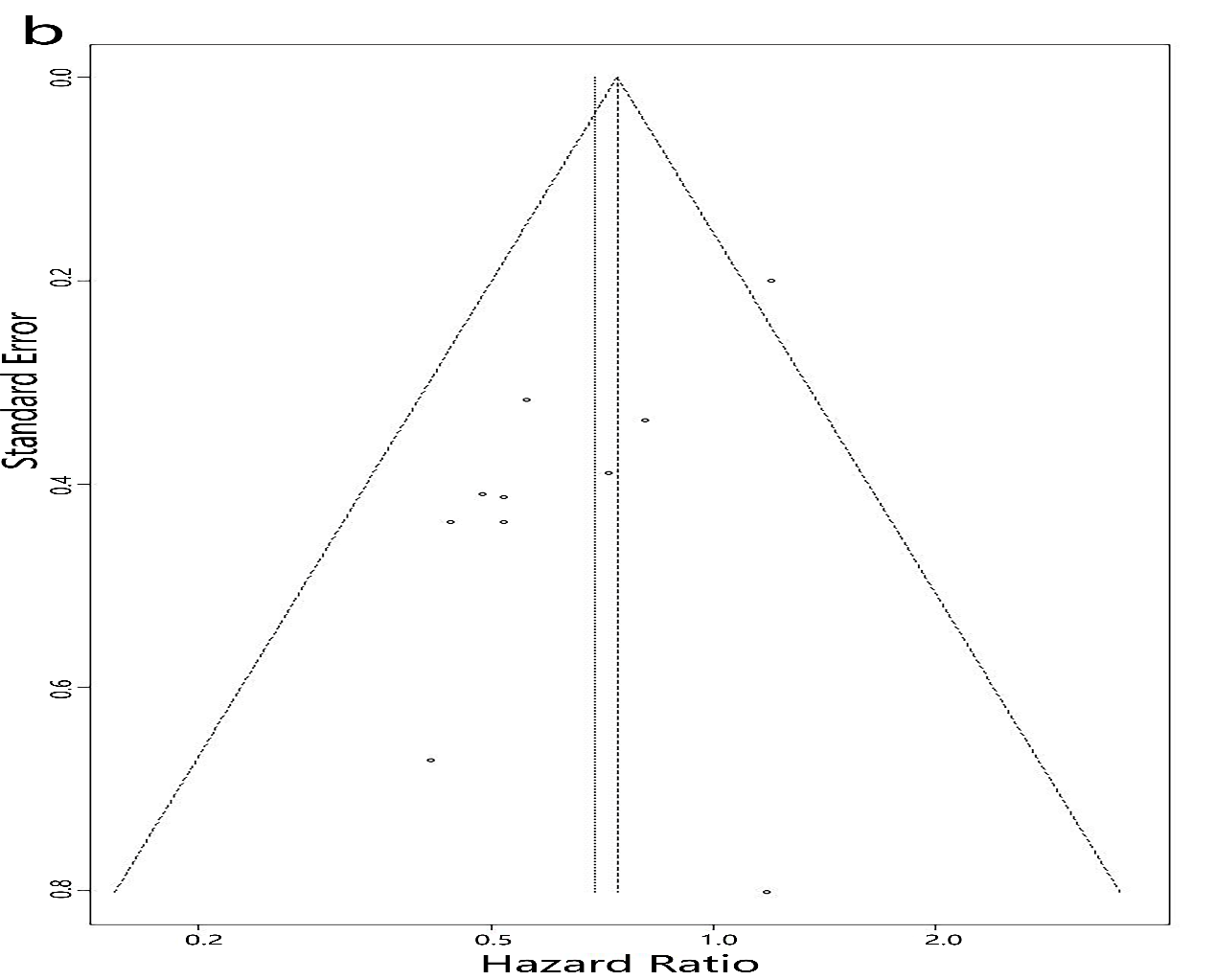

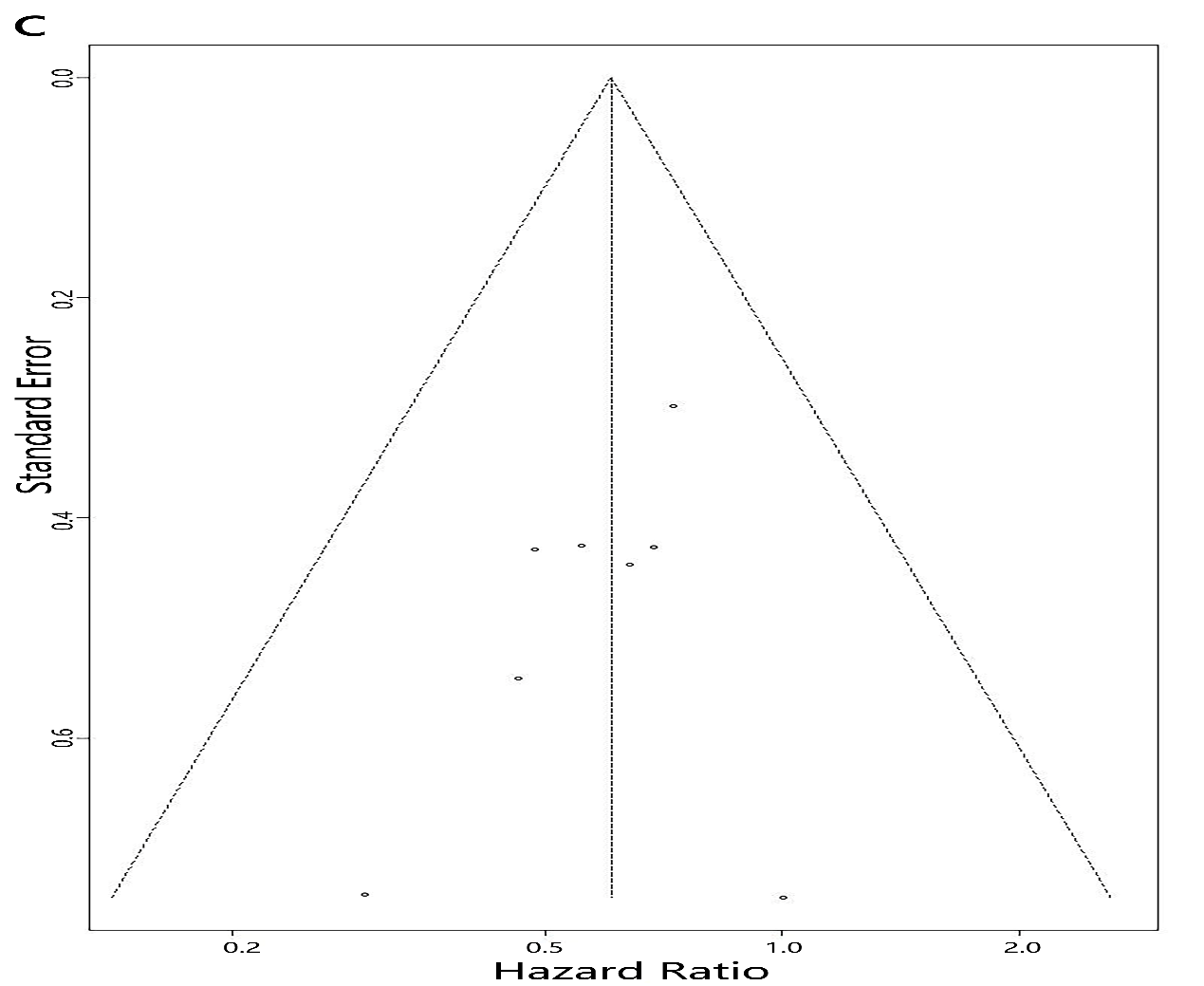

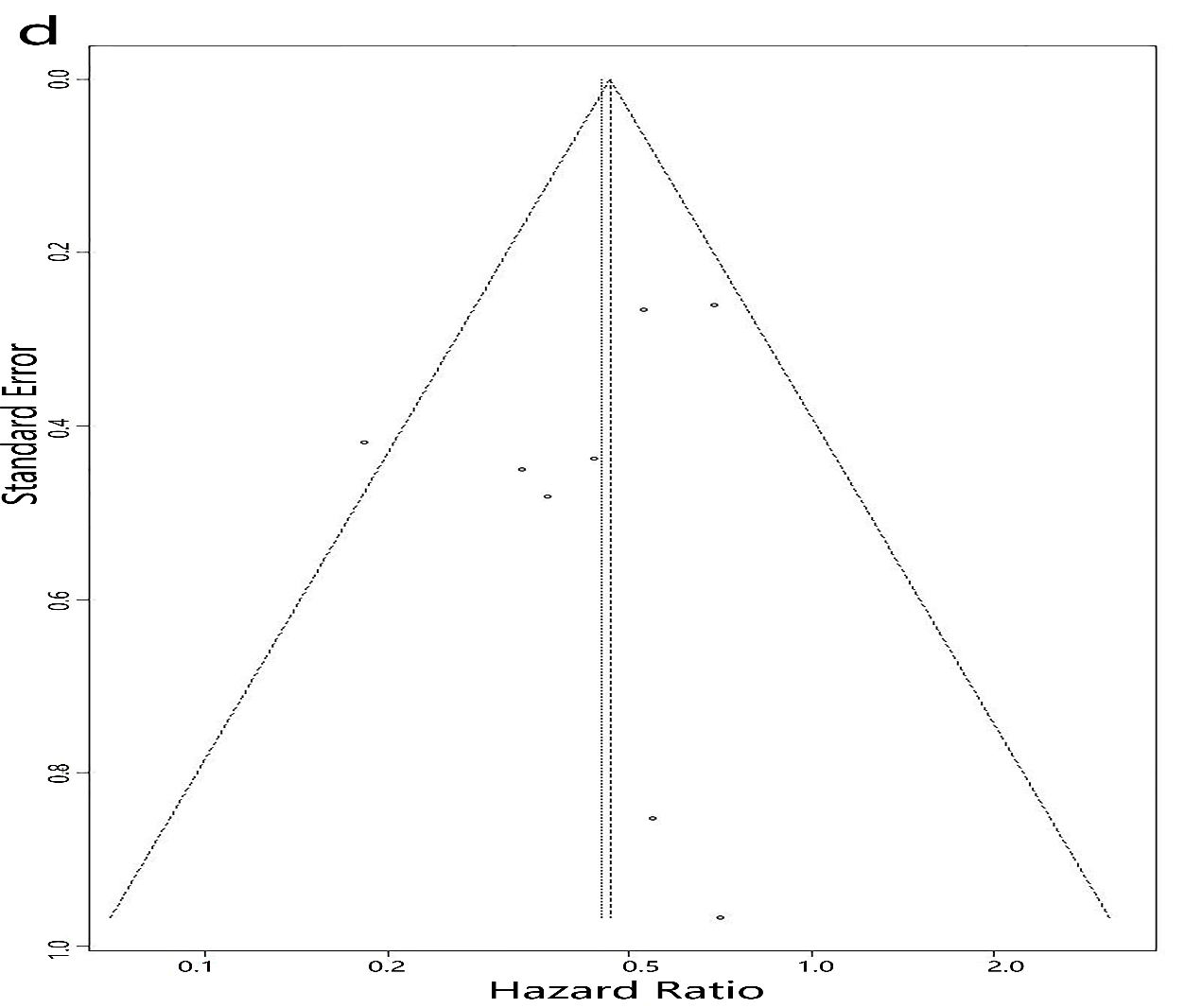


**Fig. S2** Funnel plot of (a) overall survival (OS), (b) progression-free survival (PFS), (c) disease specific survival (DSS), and (d) relapse free survival (RFS) for POLEmut compared with POLEwt EC patients.
