# Supplementary material for: The clinicopathological characteristics of POLE-mutated/ultramutated endometrial carcinoma and prognostic value of POLE status: a meta-analysis based on 49 articles incorporating 12,120 patients
Source: BMC Cancer. 2022 Nov 10;22:1157. doi: 10.1186/s12885-022-10267-2 (PMC9647950; doi:10.1186/s12885-022-10267-2)
Supplement: Supplementary file 6 — Additional file 6: Table S4. The Newcastle-Ottawa scale for quality assessment of the studies. [file 12885_2022_10267_MOESM6_ESM.docx]

**Table S4 The Newcastle-Ottawa scale for quality assessment of the studies**

| Included study | Selection | | | | Comparability | Exposure/outcome | | | Total |
| --- | --- | --- | --- | --- | --- | --- | --- | --- | --- |
|  | Is the case definition adequate? /ascertainment of exposure | Representativeness of the cases/ exposed cohort | Selection of controls/the non-exposed cohort | Definition of controls/demonstration that outcome of interest was not present at start of study | Comparability of both groups/ cohorts on the basis of the design or analysis | Ascertainment of exposure/ assessment of outcome | same method of ascertainment for both groups/ was follow-up long enough for outcomes to occur | Non-response rate/ adequacy of follow up of cohorts | scores |
| Abdulfatah E | * | * | * | * | * | * | * | * | 8 |
| Beinse G | * | * | * | * | * | * | * | * | 8 |
| Bellone S | * | * | * | * | / | * | * | * | 7 |
| Billingsley CC | * | * | * | * | ** | * | * | * | 9 |
| Bosquet JG | * | * | * | * | / | * | * | * | 7 |
| Bosse T | * | * | * | * | ** | * | * | * | 9 |
| Church DN | * | * | * | * | ** | * | * | * | 9 |
| Church DN | * | * | * | * | ** | * | * | * | 9 |
| Conlon N | * | * | * | * | / | * | * | * | 7 |
| Cosgrove CM | * | * | * | * | ** | * | * | * | 9 |
| Crumley S | * | * | * | * | / | * | * | * | 7 |
| Dai YB | * | * | * | * | ** | * | * | * | 9 |
| DeLair DF | * | * | * | * | / | * | * | * | 7 |
| Devereaux KA | * | * | * | * | ** | * | * | * | 9 |
| Eggink FA | * | * | * | * | ** | * | * | * | 9 |
| Espinosa I | * | * | * | * | / | * | * | * | 7 |
| Espinosa I | * | * | * | * | / | * | * | * | 7 |
| van Esterik M | * | * | * | * | ** | * | * | * | 9 |
| Falcone F | * | * | * | * | / | * | * | * | 7 |
| Le Gallo M | * | * | * | * | / | * | * | * | 7 |
| Haraldsdottir S | * | * | * | * | / | * | * | * | 7 |
| Haruma T | * | * | * | * | ** | * | * | * | 9 |
| He D | * | * | * | * | ** | * | * | * | 9 |
| Hoang LN | * | * | * | * | / | * | * | * | 7 |
| Imboden S | * | * | * | * | ** | * | * | * | 9 |
| Joehlin-Price A | * | * | * | * | ** | * | * | * | 9 |
| Jones NL | * | * | * | * | * | * | * | * | 8 |
| Kim SR | * | * | * | * | ** | * | * | * | 9 |
| Kolehmainen AM | * | * | * | * | ** | * | * | * | 9 |
| León-Castillo A | * | * | * | * | ** | * | * | * | 9 |
| Li YR | * | * | * | * | ** | * | * | * | 9 |
| López-Reig R | * | * | * | * | ** | * | * | * | 9 |
| McConechy MK | * | * | * | * | ** | * | * | * | 9 |
| Meng B | * | * | * | * | ** | * | * | * | 9 |
| Monsur M | * | * | * | * | ** | * | * | * | 9 |
| Da Cruz Paula A | * | * | * | * | ** | * | * | * | 9 |
| Prendergast EN | * | * | * | * | * | * | * | * | 8 |
| Riggs MJ | * | * | * | * | * | * | * | * | 8 |
| Rosa-Rosa JM | * | * | * | * | / | * | * | * | 7 |
| Siraj AK | * | * | * | * | ** | * | * | * | 9 |
| Stasenko M | * | * | * | * | / | * | * | * | 7 |
| Talhouk A | * | * | * | * | ** | * | * | * | 9 |
| Talhouk A | * | * | * | * | ** | * | * | * | 9 |
| Tessier-Cloutier B | * | * | * | * | * | * | * | * | 8 |
| Cancer Genome Atlas Research Network | * | * | * | * | / | * | * | * | 7 |
| Timmerman S | * | * | * | * | ** | * | * | * | 9 |
| Wong A | * | * | * | * | ** | * | * | * | 9 |
| ZHANG K | * | * | * | * | / | * | * | * | 7 |
| Zong LJ | * | * | * | * | ** | * | * | * | 9 |
